# Enhanced renoprotective effect of IGF-1 modified human umbilical cord-derived mesenchymal stem cells on gentamicin-induced acute kidney injury

**DOI:** 10.1038/srep20287

**Published:** 2016-02-02

**Authors:** Pengfei Liu, Yetong Feng, Delu Dong, Xiaobo Liu, Yaoyu Chen, Yi Wang, Yulai Zhou

**Affiliations:** 1 Department of Regenerative Medicine, School of Pharmaceutical Science, Jilin University, Changchun, P.R. China; 2 Key Laboratory of Regenerative Biology, Guangdong Provincial Key Laboratory of Stem Cell and Regenerative Medicine, Guangzhou Institutes of Biomedicine and Health, Chinese Academy of Sciences, Guangzhou, P.R. China; 3 Institute of Human Virology, Zhongshan School of Medicine, Sun Yat-sen University, Guangzhou, P.R. China

## Abstract

The therapeutic action of umbilical cord-derived mesenchymal stem cells (UC-MSCs) against acute kidney injury (AKI) has been demonstrated by several groups. However, how to further enhance the renoprotective effect of UC-MSCs and improve the therapy effect, are still unclear. In this study, we mainly investigated whether insulin-like growth factor-1 (IGF-1)-modified UC-MSCs hold an enhanced protective effect on gentamicin-induced AKI *in vivo*. Our results indicated that the IGF-1 overexpression could enhance the therapeutic action of human UC-MSCs, and the AKI rats treated with IGF-1-overexpressed UC-MSCs (UC-MSCs-IGF-1) showed better recovery of biochemical variables in serum or urine associated with renal function, histological injury and renal apoptosis, compared with AKI rats treated with normal UC-MSCs. RNA microarray analysis indicated that some key genes in the signal pathways associated with anti-oxidation, anti-inflammatory, and cell migratory capacity were up-regulated in UC-MSCs-IGF-1, and the results were further confirmed with qPCR. Furthermore, a series of detection *in vitro* and *in vivo* indicated that the UC-MSCs-IGF-1 hold better anti-oxidation, anti-inflammatory, and cell migratory capacity for IGF-1 overexpression. Thus, our study indicated that enhancement of UC-MSCs bioactivities with IGF-1 overexpression could increase the UC-MSCs therapeutic potential and further developed a new therapeutic strategy for the treatment of AKI.

Acute kidney injury (AKI) is considered a common disease that accounts for 2–15% of hospitalized patients. The clinical symptoms of AKI manifest as a rapid loss of the ability of the kidney to concentrate urine, excrete wastes, and maintain fluid and electrolyte homeostasis^1^,^2^,^3^. Although advances have been made to improve the therapy effect of AKI in recent years, this disease remains a high risk factor for morbidity and mortality^4^,^5^. Current clinical therapeutic options for AKI are limited to the application of supportive measures and dialysis. However, some scientists found that several therapeutic agents used in clinical practice could produce functional impairment and kidney injury, and supportive measures also required patients to wait for renal function to recover^6^,^7^,^8^. Therefore, a novel therapeutic strategy should be developed to ameliorate the survival outcomes of patients with AKI.

In recent years, stem cell-based therapy mode has been used in kinds of diseases treatment gradually, such as diabetes^9^, neural disease^10^,^11^, and so on^12^,^13^. For the treatment of AKI, several stem cell-based therapy modes have been established by scientists, and different types of stem cells, such as hematopoietic progenitor cells^14^, amniotic fluid stem cells^15^, adipose-derived stem cells^5^, even induced pluripotent stem cells^16^, have been investigated and determined to hold therapeutic effects against AKI. Especially for the action of umbilical cord-derived mesenchymal stem cells (UC-MSCs), several studies have used UC-MSCs to treat AKI in different animal models and their results indicated that renal function and structure could be improved with the infusion of UC-MSCs^3^,^17^,^18^,^19^. Compared with other mesenchymal stem cells, UC-MSCs exhibit higher frequency of colony-forming unit fibroblast and mutilineage differentiation potential without controversy^20^. Besides, UC-MSCs also hold the potential to be applied in allogeneic transplantation without obvious immune rejection, for their immunomodulatory ability and low immunogenicity^21^,^22^,^23^.

In 2013, Chen K *et al*. evaluated the organ bio-distributions of transplanted mesenchymal stem cells in mice with cisplatinum-induced AKI, and found that the cells were largely localized in pulmonary capillaries after intravenous administration. Moreover, only a minute fraction of transplanted cells could enter the kidneys, exhibiting transient survival. Therefore, the development of novel strategies in enhancing cell homing to target tissues is regarded as a prerequisite for the success of stem cell-based systemic therapies. In recent years, scientists have established several methods to enhance stem cell migration in AKI model^24^,^25^,^26^. For example, Xinaris *et al*. showed that the preconditioning of bone marrow-derived mesenchymal stem cell with insulin-like growth factor-1 (IGF-1) before infusion could improve cell migration, and this study also indicated that promoting stem cell migration could increase their therapeutic potential against AKI^24^. Interestingly, IGF-1 also has been demonstrated to hold other distinct effects that underlie the reparative actions of mesenchymal stem cells by scientists^27^. This factor secreted by mesenchymal stem cells has been reported to significantly contribute to therapeutic effect by its proliferative and anti-apoptotic actions^28^. However, whether those biological functions still exist for UC-MSCs in AKI model remains unclear.

In this study, IGF-1-overexpressed UC-MSCs (UC-MSCs-IGF-1) were established through retroviral infection, and the renoprotective effect of UC-MSCs-IGF-1 on gentamicin-induced AKI was evaluated in nude rats. Our results indicated that this method was a feasible strategy in promoting UC-MSCs migration, anti-oxidation, anti-inflammatory, and anti-apoptosis capacity to repair kidney tissues in the rat model of AKI. Therefore, this strategy could be used to develop a novel treatment mode for the preclinical study of AKI.

## Results

### Characterization of human UC-MSCs

**Figure 1:**
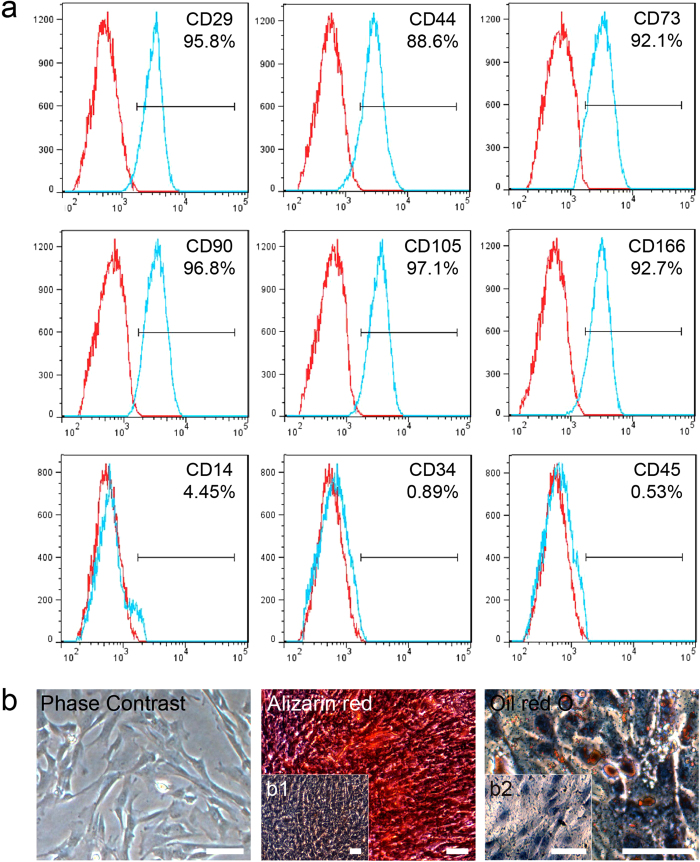
Characterization of UC-MSCs. (**a**) Immunophenotype of isolated UC-MSCs. Isolated UC-MSCs were characterized by FACS. UC-MSCs were positive for CD29, CD44, CD73, CD90, CD105, and CD166, and nearly negative for CD14, CD34, and CD45. (**b**) Differentiation characteristics of UC-MSCs. The phase contrast of UC-MSCs is shown on the left. Osteogenic differentiation was detected by Alizarin red staining (middle), and adipogenic differentiation was visualized by Oil Red O staining of the lipid vesicles (right). UC-MSCs cultured with normal medium were used as the negative control group for each stain (b1 and b2). All scale bars correspond to 100 μm.

The UC-MSCs isolated from human umbilical cord were characterized by FACS for CD markers. The results indicated that the UC-MSCs used in our study were positive for CD29 (95.8%), CD44 (88.6%), CD73 (92.1%), CD90 (96.8%), CD105 (97.1%), and CD166 (92.7%), and nearly negative for CD14 (4.45%), CD34 (0.89%) and CD45 (0.53%), which were considered as the specific markers of hematopoietic cells (Fig. 1a). In our study, the UC-MSCs displayed a spindle-shaped “fibroblast” appearance, and they were successfully differentiated into osteoblasts and adipocytes, as demonstrated by positive staining with Alizarin red and Oil red O respectively (Fig. 1b). In the negative control group, the UC-MSCs cultured with normal medium were used for each stain, and those cells could not be stained with Alizarin red and Oil red O (Fig. 1b1,b2).

### Analysis of UC-MSCs infected with human IGF-1 genes

**Figure 2:**
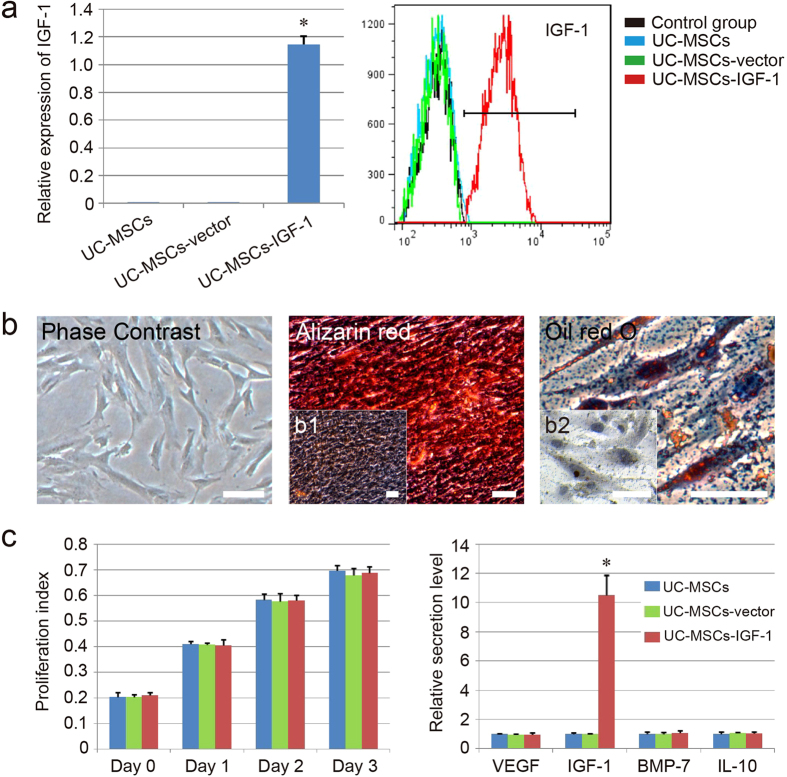
Effects of IGF-1 overexpression on the biological function of UC-MSCs. (**a**) Analysis of IGF-1 expression in different groups by qPCR (left) and FACS (right). (**b**) Differentiation characteristics of UC-MSCs-IGF-1. The phase contrast of UC-MSCs-IGF-1 is shown on the left. Osteogenic differentiation was detected by Alizarin red staining (middle), and adipogenic differentiation was visualized by Oil Red O staining of the lipid vesicles (right). UC-MSCs-IGF-1 cultured with normal medium were used as the negative control group for each stain (b1 and b2). All scale bars correspond to 100 μm. (**c**) Effect of IGF-1 overexpression on the cell proliferation and secretion. The secretion level of the normal group was regared as 1.0, and the relative secretion level of the other groups was evaluated. Results are expressed as mean ± SEM. A t-test was used to compare the various groups, and P < 0.05 was considered statistically significant. *P < 0.05 compared with the normal UC-MSCs and UC-MSCs-vector group respectively.

To assess IGF-1 expression in UC-MSCs infected with human IGF-1 genes (UC-MSCs-IGF-1), qPCR and FACS analysis were performed in our study. QPCR analysis indicated that the expression level of IGF-1 were significantly higher in UC-MSCs-IGF-1 compared with UC-MSCs and UC-MSCs-vector (UC-MSCs infected with blank vector), whereas the expression levels of IGF-1 in UC-MSCs and UC-MSCs-vector were almost undetectable (Fig. 2a). Besides, the FACS analysis revealed that the expression of IGF-1 protein was significantly up-regulated in UC-MSCs-IGF-1 (93.7%), compared with UC-MSCs (0.96%) and UC-MSCs-vector (1.43%) (Fig. 2a). These results indicated that UC-MSCs-IGF-1 were successfully generated by retroviral infection. Similar to normal UC-MSCs, the UC-MSCs-IGF-1 also displayed a spindle-shaped “fibroblast” appearance, and they could be successfully differentiated into osteoblasts and adipocytes, as demonstrated by positive staining with Alizarin red and Oil red O respectively (Fig. 2b).

In addition, we evaluated the effect of IGF-1 overexpression on the proliferation ability of UC-MSCs. The results indicated that IGF-1 overexpression in UC-MSCs did not affect cell proliferation ability *in vitro*. It was apparent that the proliferation index of UC-MSCs-IGF-1 showed no significant difference compared with that of UC-MSCs and UC-MSCs-vector (Fig. 2c). On the other side, the effects of IGF-1 overexpression on the secretory capacity of UC-MSCs in secreting renal protective cell factors (VEGF, IGF-1, BMP-7, and IL-10), were also evaluated with ELISA. As shown in Fig. 2c, only IGF-1 secreted by UC-MSCs-IGF-1 exhibited an increasing tendency, and secretory capacity of UC-MSCs-IGF-1 in secreting VEGF, BMP-7, and IL-10, did not showed obvious increase compared with that of UC-MSCs and UC-MSCs-vector.

### Evaluation of node rat AKI model

In this study, the AKI model was induced by gentamicin in adult male nude rats. To confirm the rat AKI model, urine and serum samples were collected on day 8 before treatment. The levels of N-acetyl-beta-glucosaminidase (NAG) and lysozyme (LZM) in urine, as well as creatinine and urea nitrogen in serum, were measured in the normal group and AKI model group. These indices in the model group presented significantly higher levels than those in the normal group (NAG: 37.5 ± 4.0 U/L vs. 14.5 ± 1.4 U/L; LZM: 177.5 ± 13.2 U/ml vs. 33.5 ± 2.7 U/ml; creatinine: 70.5 ± 5.7 mM vs. 22.7 ± 3.4 mM; urea nitrogen: 27.2 ± 4.0 mM vs. 10.5 ± 1.3 mM, P < 0.05). These findings demonstrated that the node rat model displayed characteristics of AKI disease (Supplementary Table 1).

### Effect of treatment on biochemical variables in urine and serum

After the treatment, LZM in urine showed a statistically significant difference between the normal group and AKI model group. Only the LZM of UC-MSCs-IGF-1 group showed a little improvement compared with other groups. The levels of urea nitrogen and NAG in urine in the treatment groups were similar to the normal levels, especially for urea nitrogen in the UC-MSCs-IGF-1 group (Supplementary Table 1). The injection of UC-MSCs, UC-MSCs-vector and UC-MSCs-IGF-1 significantly decreased NAG and creatinine in serum (P < 0.05). The creatinine level in the UC-MSCs-IGF-1 group was the most similar to that of the normal group. However, no amelioration was found in urea nitrogen in serum except the UC-MSCs-IGF-1 group (Supplementary Table 1).

### Effect of treatment on renal histology

**Figure 3:**
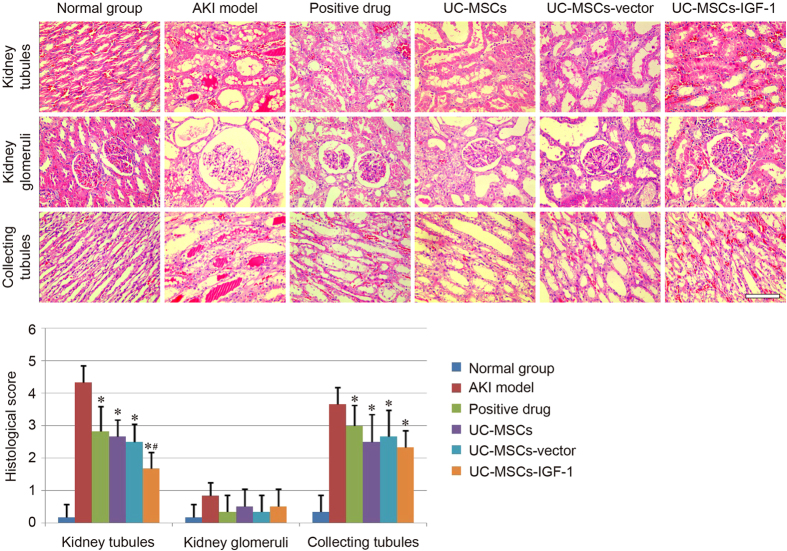
Detection of pathological changes in each group. Pathological changes in the kidney tubules, kidney glomeruli, and collecting tubules were observed under a light microscope using H&E staining. Scale bar of the phase observed through H&E staining corresponds to 100 μm. Histological score of each group was also evaluated herein. Results are expressed as mean ± SEM. A t-test was used to compare the various groups. *P < 0.05 compared with the model group. ^#^P < 0.05 compared with the normal UC-MSCs group and UC-MSCs-vector group respectively.

To evaluate the therapeutic effect of UC-MSCs, UC-MSCs-vector and UC-MSCs-IGF-1 in the AKI model, the pathological changes in the kidney tubules, kidney glomeruli, and collecting tubules of each group were observed by H&E staining. Similar to our previous studies, the typical pathological changes in AKI induced by gentamicin were mainly reflected in the kidney tubules and collecting tubules. The kidney glomeruli in all groups only showed the widening of Bowman’s space. Notable damage, including tubular necrosis, dilatation, and effusion in the kidney tubules and collecting tubules, was observed in the AKI model group compared with the normal group. Amelioration in various degrees was observed in the treatment groups, and UC-MSCs, UC-MSCs-vector and UC-MSCs-IGF-1 all showed renoprotective effect as the positive drug, and there was no difference between the UC-MSCs group and the UC-MSCs-vector group in terms of histopathology. The UC-MSCs-IGF-1 group exhibited fewer necrotic and dilated tubules and less effusion in the tubules, and the three stem cell-treated groups did not show obvious difference in the recovery of collecting tubules (Fig. 3).

A histological scoring system was further used to evaluate kidney tissue morphology. The histological score of kidney tubules in normal group was below 1, whereas that of the model group exceeded 4. The score of treatment groups could be decreased in different degrees, and the score of UC-MSCs-IGF-1 group was lower than the UC-MSCs group and the UC-MSCs-vector group respectively. However, for the histological score of kidney glomeruli and collecting tubules, there was no significant difference among three stem cell-treated groups (Fig. 3).

### Cell apoptosis assay

**Figure 4:**
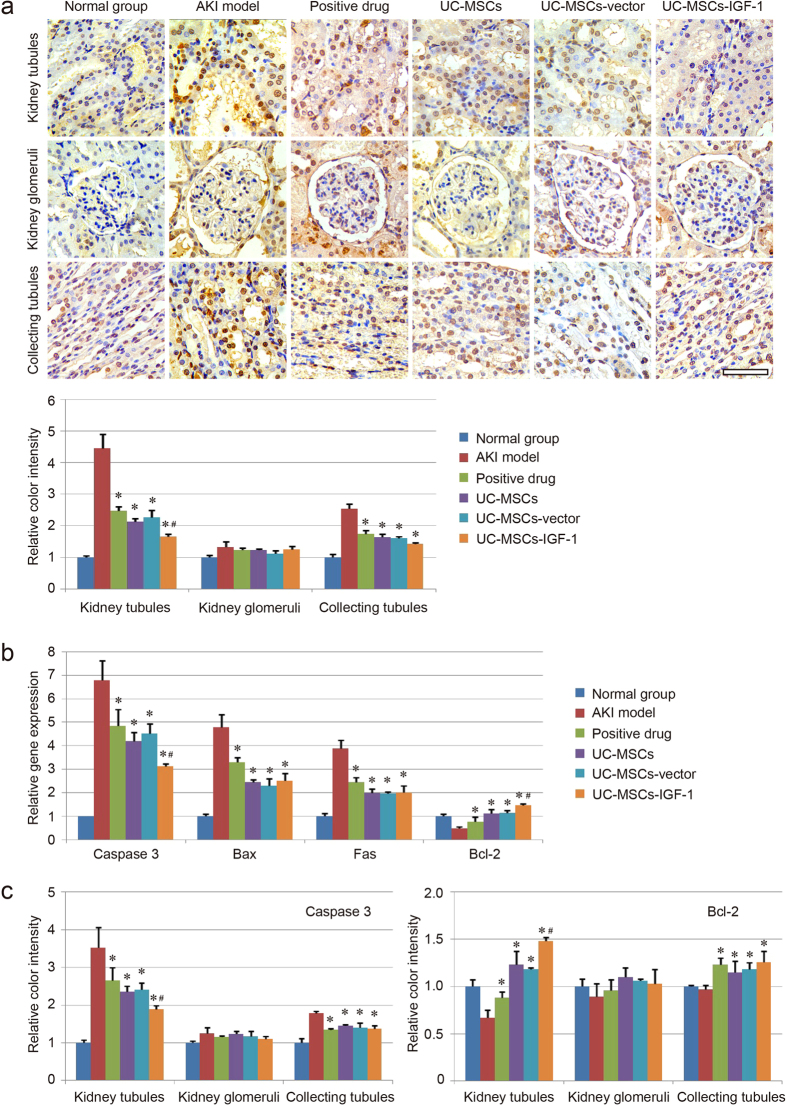
Evaluation of cell apoptosis in each group. (**a**) Images of TUNEL staining in the kidney tubules, kidney glomeruli, and collecting tubules are shown for each group. The color intensity was measured using Motic Image Advanced 3.2. The color intensity of the normal group was regarded as 1.0, and the relative color intensity of the other groups was evaluated. (**b**) Detection of apoptotic genes (Caspase 3, Bax and Fas) and anti-apoptotic gene (Bcl-2) expression using qPCR. The gene expression level in the normal group was regarded as 1.0, and the relative gene expression level of each group was further evaluated. (**c**) Detection of Caspase 3 and Bcl-2 expression using immunohistochemistry. The color intensity was further measured using Motic Image Advanced 3.2. The color intensity of the normal group was regarded as 1.0, and the relative color intensity of the other groups was further evaluated. Results are expressed as mean ± SEM. A *t*-test was used to compare the various groups, and P < 0.05 was considered statistically significant. *P < 0.05 compared with the model group; ^#^P < 0.05 compared with the normal UC-MSCs group and UC-MSCs-vector group respectively. Scale bar corresponds to 50 μm.

In our study, TUNEL assay was used to detect apoptosis in renal cells in the kidney sections. The results indicated that the number of TUNEL-positive cells in both the kidney tubules and collecting tubules markedly increased in the model group but decreased in the stem cell-treated group (Fig. 4a). Motic Image Advanced 3.2 software showed that the color intensity of the UC-MSCs-IGF-1 group was lighter than that of the UC-MSCs group and the UC-MSCs-vector group in the kidney tubules respectively. However, the UC-MSCs-IGF-1 group did not show a superior improvement in the therapeutic effect in the collecting tubules. In addition, TUNEL assay detected no significant difference in cell apoptosis of the kidney glomeruli between the model group and other groups (Fig. 4a).

The expression levels of apoptotic genes (Caspase 3, Bax, and Fas) and anti-apoptotic gene (Bcl-2) in the kidney tissue of each group were detected using real-time qPCR, and the results showed that the expression levels of those apoptotic genes in the AKI model group were much higher than those in the normal group. The stem cell-based treatments were efficient in inducing anti-apoptosis, and the UC-MSCs-IGF-1 group showed lower expression level of Caspase 3, and higher expression level of anti-apoptotic gene (Bcl-2) than those in the UC-MSCs group and the UC-MSCs-vector group (Fig. 4b). The expression levels of apoptotic gene (Caspase 3) and anti-apoptotic gene (Bcl-2) in kidney tissues were also confirmed using immunohistochemistry. A representative set close to the average level of each group was shown in Supplementary Figure 1, and the results were similar to those of qPCR. Gene expression levels in the kidney tubules, kidney glomeruli, and collecting tubules were analyzed respectively, and Motic Image Advanced 3.2 software was used to measure the color intensity of each group. No noticeable difference in gene expression was observed between the model and other groups in the kidney glomeruli. Both the positive drug and stem cell-treated groups showed a therapeutic effect on cell apoptosis in the collecting tubules. The differences among each group were mainly reflected in the kidney tubules. We found that Caspase 3 was down-regulated in the treatment groups. The UC-MSCs-IGF-1 group exhibited lower expression of apoptotic gene (Caspase 3) and higher expression of anti-apoptotic gene (Bcl-2) than those in the UC-MSCs group and the UC-MSCs-vector group (Fig. 4c). The result was similar to that of qPCR and the UC-MSCs-IGF-1 group showed better therapeutic action on inhibiting cell apoptosis (Fig. 4c).

### RNA microarray analysis of UC-MSCs, UC-MSCs-vector and UC-MSCs-IGF-1

**Figure 5:**
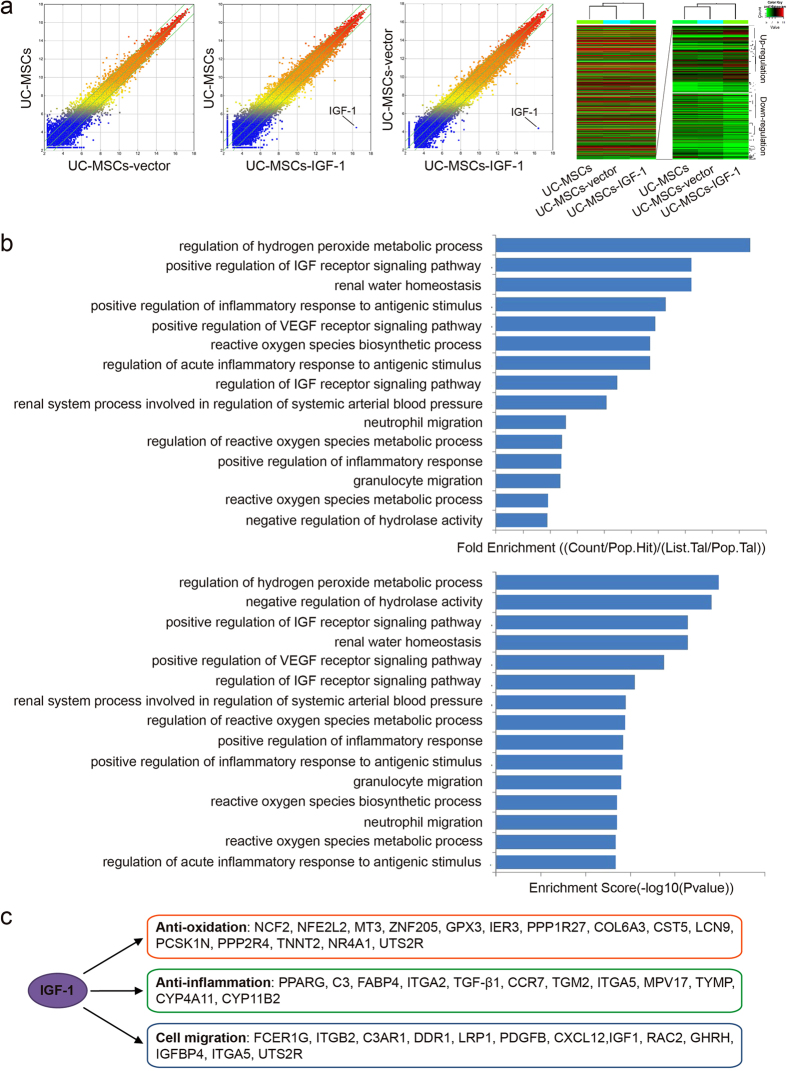
RNA microarray analysis of UC-MSCs, UC-MSCs-vector and UC-MSCs-IGF-1. (**a**) Comparison of global gene expression profiles of UC-MSCs, UC-MSCs-vector and UC-MSCs-IGF-1. The values of X and Y axes in the Scatter-Plot were normalized signal values of each sample (log2 scaled). The green lines are Fold Change Lines (The default fold change value given was 2.0). Over two fold alterations of genes between two compared samples could be found above the top green line and below the bottom green line. In Hierarchical Clustering for “all targets value”, “Red” indicates high relative expression, and “green” indicates low relative expression. The heatmap of all detected genes was shown on the left, and the heatmap of the up-regulated or down-regulated genes in UC-MSCs-IGF-1 was shown on the right. (**b**) Analysis of Gene Ontology project associated with cell migration, anti-oxidation capacity, and anti-inflammatory capacity. The bar plots showed the Fold Enrichment and Enrichment Score value of the significant enrichment terms. (**c**) Genes connected to IGF-1 overexpression through bio-informatics prediction.

Gene expression in UC-MSCs, UC-MSCs-vector and UC-MSCs-IGF-1 was verified by RNA arrays. The Scatter-Plot and Hierarchical Clustering were performed based on all target values to assess the difference in gene expression among UC-MSCs, UC-MSCs-vector and UC-MSCs-IGF-1, and the results showed that nearly one thousand genes were up-regulated or down-regulated in UC-MSCs-IGF-1 obviously, compared with UC-MSCs and UC-MSCs-vector. As the RNA microarray detected nearly thirty-five thousands genes in total, the change of less than 3% of all genes might not be obvious in the heatmap of all genes. Therefore, another heatmap of the up-regulated or down-regulated genes in UC-MSCs-IGF-1 was shown in Fig. 5a, indicating the effect of IGF-1 on the expression of other genes (the raw data of the heatmap could be found in Supplementary File 1). The clustering analysis in heatmap indicated that normal UC-MSCs and UC-MSCs-vector hold similar expression profile and their expression profiles were both different from that of UC-MSCs-IGF-1 (Fig. 5a).

The Gene Ontology project provided a controlled vocabulary to describe genes, associated with cell migration, anti-oxidation, and anti-inflammatory capacity, up-regulated in UC-MSCs-IGF-1 compared with UC-MSCs-vector. The bar plots showed the Fold Enrichment and Enrichment Score value of the significant enrichment terms associated with cell migration, anti-oxidation, and anti-inflammatory capacity respectively (Fig. 5b). On the other side, genes connected to IGF-1 overexpression through bio-informatics prediction had been represented in Fig. 5c, and those genes were classified into three groups, genes associated anti-oxidation, anti-inflammation and cell migration respectively, indicating the IGF-1 overexpression could enhance those abilities of human UC-MSCs.

The dataset of microarray analysis have been submitted in Gene Expression Omnibus, and the accession number is “GSE73724”.

### Anti-oxidation capacity assay

**Figure 6:**
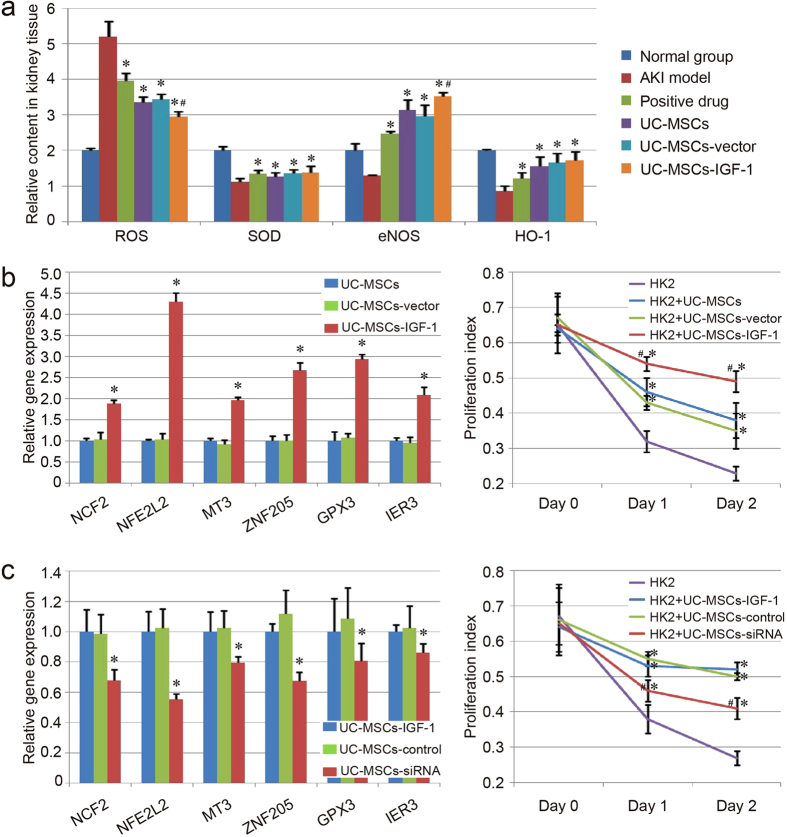
Evaluation of anti-oxidation capacity of UC-MSCs, UC-MSCs-vector and UC-MSCs-IGF-1. (**a**) Relative contents of ROS and some antioxidants (SOD, eNOS and HO-1) in injured kidney tissues of each group. The contents of ROS and those antioxidants in the normal group were regarded as 1.0, and the relative contents of the other groups were further evaluated. Results are expressed as mean ± SEM. A t-test was used to compare the various groups, and P < 0.05 was considered statistically significant. *P < 0.05 compared with the model group; #P < 0.05 compared with the normal UC-MSCs group and UC-MSCs-vector group respectively. (**b**) Evaluation of anti-oxidation capacity of UC-MSCs, UC-MSCs-vector and UC-MSCs-IGF-1 *in vitro*. Expression of the genes about anti-oxidation function (NCF2, NLE2L2, MT3, ZNF205, GPX3 and IER3) was detected with qPCR. The level of gene expression in UC-MSCs was regarded as 1.0. *P < 0.05 compared with the normal UC-MSCs group and UC-MSCs-vector group respectively. The co-culture system was established with HK-2 cells and UC-MSCs (blue line)/UC-MSCs-vector (green line) /UC-MSCs-IGF-1 (red line). The proliferation index of each co-culture group exposed to 30 μM H_2_O_2_ was determined with CCK-8. *P < 0.05 compared with the control group; ^#^P < 0.05 compared with the normal UC-MSCs group and UC-MSCs-vector group respectively. (**c**) Effect of IGF-1-siRNA on the anti-oxidation function of UC-MSCs-IGF-1. Expression of NCF2, NLE2L2, MT3, ZNF205, GPX3 and IER3 was further detected with qPCR in UC-MSCs-IGF-1, UC-MSCs-control and UC-MSCs-siRNA. The level of gene expression in UC-MSCs-IGF-1 was regarded as 1.0. The co-culture system was also established with HK-2 cells and those stem cells (UC-MSCs-IGF-1: blue line, UC-MSCs-control: green line, UC-MSCs-siRNA: red line), and the proliferation index of each co-culture group exposed to 30 μM H_2_O_2_ was determined as before.

The levels of the ROS, as well as some antioxidants (SOD, eNOS and HO-1) in injured kidney tissues were evaluated after the treatment. We found that the level of the ROS was increased in the AKI model group, and decreased in the treatment groups. A converse tendency existed in the levels of those antioxidants. The UC-MSCs-IGF-1 group exhibited lower level of ROS and higher level of the antioxidant (eNOS) than those in the UC-MSCs group and the UC-MSCs-vector group (Fig. 6a). The results indicated that the treatment with UC-MSCs-IGF-1 hold better anti-oxidation potential than the treatment with UC-MSCs or UC-MSCs-vector.

Based on the prediction of RNA microarray, the expression of some genes associated with anti-oxidation was further analyzed with qPCR, and the result was similar to RNA microarray. The expression of NCF2, NLE2L2, MT3, ZNF205, GPX3 and IER3, were upregulated in UC-MSCs-IGF-1 compaerd with UC-MSCs and UC-MSCs-vector (P < 0.05), and no significant difference existed between UC-MSCs and UC-MSCs-vector (Fig. 6b). The result of co-culture experiment also indicated that HK2 cells co-cultured with UC-MSCs-IGF-1 showed best survival rate compared with other group, even with modest decreased proliferation index (Fig. 6b).

In addition, a rescue experiment was performed with IGF-1-siRNA in UC-MSCs-IGF-1, to further confirm the anti-oxidation function of IGF-1 overexpression in UC-MSCs. Herein, qPCR and FACS analysis were also performed to assess IGF-1 expression in UC-MSCs-IGF-1 treated with IGF-1-siRNA (UC-MSCs-siRNA). The result of qPCR analysis indicated that the expression of IGF-1 was significantly down-regulated in UC-MSCs-siRNA compared with UC-MSCs-IGF-1 and UC-MSCs-IGF-1 treated with nontargeting control siRNA (UC-MSCs-control) (Supplementary Figure 2a). Besides, the FACS results showed that the expression level of IGF-1 protein was significantly lower in UC-MSCs-siRNA (19.9%), compared with UC-MSCs-IGF-1 (91.6%) and UC-MSCs-control (92.1%) (Supplementary Figure 2b). These results indicated that the IGF-1-siRNA was effective in our study. In UC-MSCs-siRNA, the qPCR result showed the downregulated expression of those genes about anti-oxidation function (NCF2, NLE2L2, MT3, ZNF205, GPX3 and IER3) in different degrees compared with UC-MSCs-IGF-1 and UC-MSCs-control (P < 0.05), and the co-culture experiment also revealed the decreased survival ability of HK-2 cells co-cultured with UC-MSCs-siRNA, indicating the effect of IGF-1 on the anti-oxidation ability of UC-MSCs (Fig. 6c).

### Inflammatory components assay

**Figure 7:**
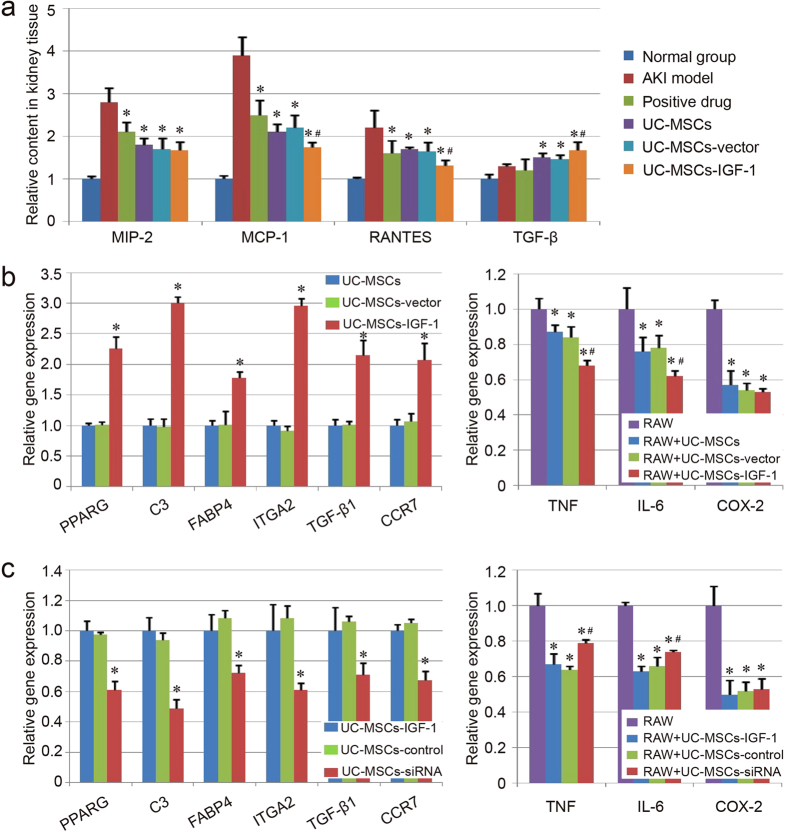
Evaluation of anti-inflammatory capacity of UC-MSCs, UC-MSCs-vector and UC-MSCs-IGF-1. (**a**) Detection of inflammatory component using ELISA. The contents of the inflammatory components in the normal group were regarded as 1.0, and the relative contents of the other groups were further evaluated. Results are expressed as mean ± SEM. A *t*-test was used to compare the various groups, and P < 0.05 was considered statistically significant. *P < 0.05 compared with the model group; ^#^P < 0.05 compared with the normal UC-MSCs group and UC-MSCs-vector group respectively. (**b**) Evaluation of anti-inflammatory capacity of UC-MSCs, UC-MSCs-vector and UC-MSCs-IGF-1 *in vitro*. Expression of the genes about anti-inflammatory capacity (PPARG, C3, FABP4, ITGA2, TGF-β1 and CCR7) was detected with qPCR. The level of gene expression in UC-MSCs was regarded as 1.0. *P < 0.05 compared with the normal UC-MSCs group and UC-MSCs-vector group respectively. The co-culture system was established with RAW264.7 cells (RAW) and UC-MSCs/UC-MSCs-vector/UC-MSCs-IGF-1. The expression of genes associated with inflammatory response (TNF, IL-6 and COX-2) was determined with qPCR. *P < 0.05 compared with the control group; ^#^P < 0.05 compared with the normal UC-MSCs group and UC-MSCs-vector group respectively. (**c**) Effect of IGF-1-siRNA on the anti-inflammatory capacity of UC-MSCs-IGF-1. Expression of PPARG, C3, FABP4, ITGA2, TGF-β1 and CCR7 was further detected with qPCR in UC-MSCs-IGF-1, UC-MSCs-control and UC-MSCs-siRNA. The level of gene expression in UC-MSCs-IGF-1 was regarded as 1.0. The co-culture system was also established with RAW264.7 cells and those stem cells, and expression of TNF, IL-6 and COX-2, was determined with qPCR as before.

Relative contents of MCP-1, IL-10, RANTES and MIP-2 in kidney tissue were examined using ELISA in our study. The inflammatory components, MIP-2, MCP-1 and RANTES were down-regulated with the therapy in different degrees, while the anti-inflammatory component showed a little increase in the stem cell-treated group, compared with the model group (Fig. 7a). Compared with the UC-MSCs group and the UC-MSCs-vector group, our results indicated that the UC-MSCs-IGF-1 group hold better effect on down-regulating MCP-1 and RANTES, as well as up-regulating TGF-β (Fig. 7a). The inflammatory cells infiltration of each group was also evaluated in kidney tissues. Even though some inflammatory cells could be found in the renal interstitium of our AKI model group, the areas of obvious inflammatory cells infiltration were still limited. After the treatment, few obvious inflammatory cells infiltration could be observed in the HE staining of each group (Fig. 3). This may be due to the immunodeficient animals used in our study, which only have limited leukomonocytes.

Based on the bio-informatics prediction, the expression of some genes associated with regulation of inflammatory response was further evaluated in UC-MSCs, UC-MSCs-vector and UC-MSCs-IGF-1, and qPCR result indicated that the expression of PPARG, C3, FABP4, ITGA2, TGF-β1 and CCR7, were up-regulated in UC-MSCs-IGF-1 compared with UC-MSCs and UC-MSCs-vector (P < 0.05), and no significant difference could be observed between UC-MSCs and UC-MSCs-vector (Fig. 7b), which was also in agreement with RNA microarray. The result of co-culture experiment also indicated that the expression of inflammatory components, TNF, IL-6 and COX-2, in RAW264.7 cells could be down-regulated when RAW264.7 cells were co-cultured with UC-MSCs, UC-MSCs-vector and UC-MSCs-IGF-1. Besides, UC-MSCs-IGF-1 showed enhanced function to regulate the expression of TNF and IL-6 compared with normal UC-MSCs and UC-MSCs-vector (Fig. 7b).

The genes associated with regulation of inflammatory response were also evaluated in UC-MSCs-siRNA using qPCR. We could found that those genes (PPARG, C3, FABP4, ITGA2, TGF-β1 and CCR7) were down-regulated in UC-MSCs-siRNA compared with UC-MSCs-IGF-1 and UC-MSCs-control. In addition, the function to suppress inflammatory response was also attenuated in UC-MSCs-siRNA, and the expression of TNF and IL-6 was up-regulated in UC-MSCs-siRNA significantly compared with UC-MSCs-IGF-1 and UC-MSCs-control, even though the UC-MSCs-siRNA still could inhibit the inflammatory components expression in RAW264.7 cells (Fig. 7c).

### Cell migration assay

**Figure 8:**
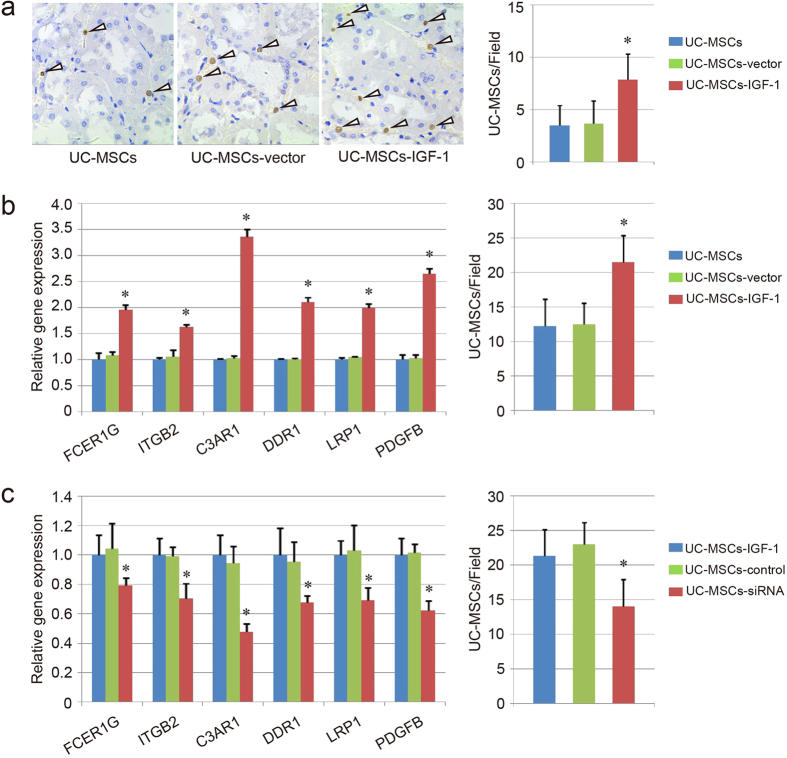
Evaluation of migratory capacity of UC-MSCs, UC-MSCs-vector and UC-MSCs-IGF-1. (**a**) Migratory capacity of UC-MSCs, UC-MSCs-vector and UC-MSCs-IGF-1 *in vivo*. Representative images of hNA-positive cells in the UC-MSCs group, UC-MSCs-vector group and UC-MSCs-IGF-1 group. The number of BrdU-positive cells was further counted in each field. (**b**) Evaluation of migratory capacity of UC-MSCs, UC-MSCs-vector and UC-MSCs-IGF-1 *in vitro*. Expression of the genes about migratory capacity (FCER1G, ITGB2, C3AR1, DDR1, LRP1 and PDGFB) was detected with qPCR. The level of gene expression in UC-MSCs was regarded as 1.0. The cell migratory capacity was further evaluated using transfilter assay. The number of migrating cells was counted in each field. *P < 0.05 compared with the normal UC-MSCs group and UC-MSCs-vector group respectively. (**c**) Effect of IGF-1-siRNA on the migratory capacity of UC-MSCs-IGF-1. Expression of FCER1G, ITGB2, C3AR1, DDR1, LRP1 and PDGFB was further detected with qPCR in UC-MSCs-IGF-1, UC-MSCs-control, UC-MSCs-siRNA. The level of gene expression in UC-MSCs-IGF-1 was regarded as 1.0. The transfilter assay was also performed to determine the cell migratory capacity as before.

To investigate the effect of IGF-1 overexpression on UC-MSCs, and explain the improved therapeutic effect in the UC-MSCs-IGF-1 group, the migratory ability of UC-MSCs-IGF-1 was detected *in vivo* and *in vitro*. To evaluate migration *in vivo*, anti-human nucleus antigen (hNA), an antigen associated with the nuclei in human cells, was used to detect the location of human UC-MSCs in kidney tissue with immunohistochemistry, and the result indicated that the UC-MSCs-IGF-1 group had more positive cells than the UC-MSCs group and the UC-MSCs-vector group (Fig. 8a).

The expression of genes associated with cell migration, showing obvious change in RNA microarray, was further evaluated using qPCR. The result indicated that the expression of FCER1G, ITGB2, C3AR1, DDR1, LRP1 and PDGFB, were upregulated in UC-MSCs-IGF-1 compared with UC-MSCs and UC-MSCs-vector (P < 0.05), and no significant difference could be observed between UC-MSCs and UC-MSCs-vector (Fig. 8b), which was similar to the result of RNA microarray. In addition, an *in vitro* injury-migration model was applied based on a transwell system consisting of UC-MSCs co-cultured with cisplatin-injured HK2 cells. UC-MSCs-IGF-1 migration from the upper chamber across the membrane to the cisplatin-damaged HK2 cells could be enhanced compared with normal UC-MSCs and UC-MSCs-vector (Fig. 8b).

The expression of FCER1G, ITGB2, C3AR1, DDR1, LRP1 and PDGFB was also evaluated in UC-MSCs-siRNA. The result showed that IGF-1-siRNA could down-regulate the expression of those genes associated with cell migration in UC-MSCs-IGF-1. Besides, the transwell migration system also confirmed the result, that the migratory ability of UC-MSCs-siRNA was weaker than UC-MSCs-IGF-1 and UC-MSCs-control (Fig. 8c).

## Discussion

As early as 2010, Cao H *et al*. first demonstrated that human UC-MSCs could ameliorate ischemia/reperfusion-induced AKI in Sprague-Dawley rats, and laid the foundation for further study on the potential application of UC-MSCs in human kidney disease^17^. Later, some groups further confirmed the therapeutic action of UC-MSCs against kidney injury^19^,^29^. However, all of the studies were performed in rats or mice with normal immune system, and both the therapeutic function of UC-MSCs and the associated therapy mechanism might be influenced for immune rejection, even though the UC-MSCs hold immunomodulatory ability and low immunogenicity. To evaluate the therapeutic effect of UC-MSCs accurately, Fang TC *et al*. demonstrated that human UC-MSCs could improve renal function in immune deficiency (NOD-SCID) mice suffering from AKI^3^. In our study, the node rats were used for AKI model, which was always established with Wistar or Sprague-Dawley rats, not mice. Maybe this model was more suitable to evaluate the therapeutic action of human stem cells against AKI *in vivo*.

Even though the therapeutic effect of human UC-MSCs against AKI has been confirmed for several years, the optimizations of the UC-MSCs-based therapy in renal repair are still limited. Chen Y, *et al*. investigated the therapeutic effects of hepatocyte growth factor (HGF) modified UC-MSCs in ischemia/reperfusion-induced AKI rat models, and the results indicated that the HGF modification could promote the amelioration of ischemia/reperfusion- induced rat renal injury via anti-apoptotic and anti-inflammatory mechanisms^19^. However, the cell migratory ability was not evaluated in their research. IGF-1 can mediate many of the actions of growth hormone (GH), and both GH excess and deficiency are associated with perturbed kidney function^27^. Besides, the effects of administering IGF-1 have been examined in AKI model, and its therapeutic function against AKI has been determined by some groups as well^30^,^31^,^32^,^33^. Therefore, the enhancement of IGF-1 expression and secretion in UC-MSCs is a feasible strategy to improve the therapeutic action of UC-MSCs against AKI. On the other side, IGF-1 has also been demonstrated to hold the potential to improve migratory ability of bone marrow-derived mesenchymal stem cells. In our study, UC-MSCs-IGF-1 also showed stronger migratory ability, compare with normal UC-MSCs and UC-MSCs-vector. Besides, we also found that the anti-oxidation capacity and the anti-inflammatory capacity were enhanced with the overexpression of IGF-1 in UC-MSCs. Therefore, we can conclude that the optimization of the UC-MSCs-based therapy is not only due to the therapeutic action of IGF-1 against AKI, but also the biological effect of IGF-1 overexpression on UC-MSCs.

Our results indicated that the IGF-1 overexpression could improve cell migratory ability of UC-MSCs, while the mechanism was different from other report. In Xinaris’ study, CXCR4 could be up-regulated by IGF-1 treatment in bone marrow-derived mesenchymal stem cells, and this was considered as the key mechanism of enhanced cell migration in their research^24^. Our group also confirmed the important role of CXCR4 and CXCR7 expression for cell migratory ability in the previous study^26^. However, the expression of CXCR4 and CXCR7, detected in this study using RNA microarray and qPCR, did not show significant difference among UC-MSCs, UC-MSCs-vector, and UC-MSCs-IGF-1 (data not shown). We hypothesized that the IGF-1 might affect migratory ability of the mesenchymal stem cell from different origin through different signaling pathways. For example, PI3K-AKT signaling pathway has been demonstrated to hold the potential to regulate the migratory ability of mesenchymal stem cell^25^,^34^,^35^. In our study, we also found that the some genes in PI3K-AKT signaling pathway were up-regulated in UC-MSCs-IGF-1 (Supplementary Figure 3a), compared with UC-MSCs-vector. Besides, some genes in Leukocyte transendothelial migration pathway were also activated in UC-MSCs-IGF-1 (Supplementary Figure 3b). As we know, PI3K-AKT pathway belongs to IGF-1 regulated downstream pathway. Therefore, IGF-1/PI3K/AKT signaling pathway may hold significant position in regulating cell migratory ability. We also detected the expression of PI3K and AKT. The results indicated that the two genes were indeed up-regulated in UC-MSCs-IGF-1, compared with normal UC-MSCs and UC-MSCs-vector (Supplementary Figure 3a). However, the levels of gene upregulation were not so significant as that of IGF-1. Therefore, the associated pathway may still need our investigation with other methods. Herein, the cell apoptosis in kidney tissue of UC-MSCs-IGF-1 group showed relieved in some degree. However, the Gene Ontology project in RNA microarray indicated that the IGF-1 overexpression did not affect anti-apoptosis capacity of UC-MSCs directly. Therefore, we hypothesized that the relief of cell apoptosis in the UC-MSCs-IGF-1 group was mainly due to the therapeutic action of overexpressed IGF-1 against AKI, or the secondary effect of the enhanced anti-oxidation capacity and the anti-inflammatory capacity.

Gentamicin, a kind of aminoglycoside antibiotic, is always used to treat many types of bacterial infections. However, it is also regarded as a well-known cause of nephrotoxicity, and mainly characterized by loss of renal function, which remains a major problem in clinical application^36^. Besides, the use of toxic antibiotics is increasing due to antimicrobial resistance, especially in intensive care units. Thus, new strategies are significant to treat or prevent gentamicin-induced nephrotoxicity. In recent years, gentamicin-induced AKI model has been investigated by some groups, to develop novel therapy methods for antibiotic-induced nephrotoxicity^26^,^36^,^37^,^38^,^39^. Different from other AKI models (e.g. ischemia/reperfusion injury, cisplatin nephrotoxicity injury, and so on), the typical pathological changes in gentamicin-induced AKI were mainly reflected in the kidney tubules and collecting tubules, and the kidney glomeruli did not show obvious damage. However, the detailed mechanisms about gentamicin-induced AKI still need our further research.

Our research indicated that IGF-1 hold the potential to enhance therapeutic action of UC-MSCs against AKI through promoting anti-oxidation, anti-inflammatory, and cell migratory capacity through the studies *in vitro* and *in vivo*. The *in vitro* model should link to *in vivo* animal model clearly. At first, our group tried to establish the *in vitro* models with gentamicin, to evaluate anti-oxidation, anti-inflammatory, and cell migratory capacity of gene modified UC-MSCs. However, the experiment results indicated that gentamicin could not affect HK-2 cell proliferation or induce inflammation in RAW264.7 cells obviously, even in high concentration (Supplementary Figure 4a-4b). Our previous work also indicated that gentamicin-treated renal cells could not induce cell migration in the trans-well system^26^. Therefore, we had to establish the *in vitro* models as other references using LPS, H2O2 and cisplatin respectively^24^,^40^,^41^. Even though those models *in vitro* may not link to *in vivo* animal model clearly, they were still effective to evaluate anti-oxidation, anti-inflammatory, and cell migratory capacity of gene modified UC-MSCs.

The therapeutic mechanism of mesenchymal stem cells against AKI comprises both differentiation-dependent mechanism and differentiation-independent mechanism^42^,^43^,^44^. However, which of these two mechanisms is more significant for the therapeutic action of UC-MSCs against AKI remains unclear. Even though our results have indicated that UC-MSCs could migrate into kidney tissue, most cells are located in blood vessel or adhere on blood vessel wall in kidney, and few UC-MSCs could join into kidney tissue repair or regeneration. Therefore, whether UC-MSCs can differentiate into renal cells and further repair the damaged tissue still needs our further exploration in AKI model.

In UC-MSCs-IGF-1, some genes associated with those biological functions were activated in different degrees. However, we are still unclear which genes are regulated by IGF-1 directly, and those genes should be the key points for us to understand the biological function of IGF-1 on stem cell-based AKI therapy intensively. Therefore, maybe the detailed analysis should be performed for gene expression and signaling pathway assay associated with IGF-1 overexpression, and the enhanced understanding of the regulating mechanism of IGF-1 in different kinds of cells, may help us to develop the novel stem cell source for AKI therapy with gene regulation.

In conclusion, our study developed a novel strategy to enhance the therapeutic effect of UC-MSCs against gentamicin-induced AKI through IGF-1 overexpression. Compared with normal UC-MSCs and UC-MSCs-vector, UC-MSCs-IGF-1 hold more activity in anti-oxidation, anti-inflammation and cell migration. Therefore, the combination of stem cell-based therapy and gene regulation hold special potential in the treatment of patients with AKI in clinic, even though the detailed mechanism still need our further investigation.

## Methods

This study was carried out in strict accordance with the recommendations in the Guide for the Care and Use of Laboratory Animals of the National Institutes of Health. The protocol was approved by the Committee on the Ethics of Animal Experiments and Human Subject Research of School of Pharmaceutical Science, Jilin University. All operations were performed under sodium pentobarbital anesthesia, and all efforts were made to minimize suffering. The participants have provided the written informed consent to participate in this study.

The detailed information about the materials and methods can be found in Supplementary File 2.

## Additional Information

**How to cite this article**: Liu, P. *et al*. Enhanced renoprotective effect of IGF-1 modified human umbilical cord-derived mesenchymal stem cells on gentamicin-induced acute kidney injury. *Sci. Rep*. **6**, 20287; doi: 10.1038/srep20287 (2016).

## Supplementary Material

Supplementary Information
