# Supplementary material for: Enhanced renoprotective effect of IGF-1 modified human umbilical cord-derived mesenchymal stem cells on gentamicin-induced acute kidney injury
Source: Sci Rep. 2016 Feb 2;6:20287. doi: 10.1038/srep20287 (PMC4735814; doi:10.1038/srep20287)
Supplement: Supplementary Information [file srep20287-s1.pdf]

Enhanced renoprotective effect of IGF-1 modified human umbilical cord-derived mesenchymal stem cells on gentamicin-induced acute kidney injury

Pengfei Liu [1,2], Yetong Feng [3], Delu Dong [1], Xiaobo Liu [1,2], Yaoyu Chen [1,2], Yi Wang [1], Yulai Zhou [1*]

1. Department of Regenerative Medicine, School of Pharmaceutical Science, Jilin University, Changchun, P.R. China

2. Key Laboratory of Regenerative Biology, Guangdong Provincial Key Laboratory of Stem Cell and Regenerative Medicine, Guangzhou Institutes of Biomedicine and Health, Chinese Academy of Sciences, Guangzhou, P.R. China

3. Institute of Human Virology, Zhongshan School of Medicine, Sun Yat-sen University, Guangzhou, P.R. China

* Correspondence to: Yulai Zhou (zhouyl@jlu.edu.cn, +86-431-8561-9299)

Department of Regenerative Medicine,

School of Pharmaceutical Science,

Jilin University,

No.1266, Fujin Road, Changchun, P.R. China

Supplementary File 1

Genes up-regulated in UC-MSCs-IGF-1

ProbeName	GeneSymbol	UC-MSCs	UC-MSCs-vector	UC-MSCs-IGF-1
A_23_P43504	ABCA2	143.00017	144.9118	324.24133
A_23_P82249	ABCB8	111.96739	105.19628	228.14723
A_33_P3376947	ABCB9	33.770687	33.0744	67.897484
A_23_P128094	ABCB9	33.542255	43.84322	118.340515
A_23_P72584	ACBD7	362.07004	240.36584	791.1804
A_33_P3250730	ACCN4	760.10706	626.6783	1520.4312
A_23_P138881	ACTN3	980.22864	753.5732	1785.7445
A_33_P3864411	ACTR3BP5	2410.9546	1120.9977	9312.506
A_23_P115011	ADAMTSL4	40.150295	49.596252	121.02768
A_33_P3242383	AFF1	8.383349	9.48024	14.392249
A_33_P3253628	AGXT2L2	38.195442	32.53493	69.54955
A_33_P3368560	AHSA2	339.60925	297.68933	842.3194
A_33_P3260053	AIF1L	840.24005	745.17834	2082.4993
A_33_P3238433	ALDH3A1	40.597485	35.37849	65.37298
A_24_P131580	ALPPL2	1037.5648	850.5033	2010.5139
A_33_P3291279	ANKRD56	4.9999995	15.662179	58.75549
A_33_P3278455	ANKRD6	7.2751503	7.164659	14.865142
A_32_P148345	ANXA2	4853.517	2675.2837	10414.334
A_24_P87931	APOL1	1212.4629	1217.6804	3086.375
A_23_P73511	ARAF	585.19934	486.83337	1193.5731
A_24_P33895	ATF3	19.885704	27.230991	48.695602
A_24_P307626	ATP1A4	700.2628	728.724	1525.9015
A_23_P146146	ATP6V0D2	8.045739	6.7342277	18.346968
A_24_P276932	ATP6V1C2	183.98972	158.33847	375.15375
A_33_P3282978	BAALC	188.81126	168.63252	641.60706
A_33_P3285470	BAI1	32.718124	29.61538	54.659824
A_33_P3251932	BCL2L11	304.18738	213.02867	821.37335
A_32_P69849	BEX5	35.98655	32.56157	295.44547
A_24_P57898	BHLHE23	1148.1241	941.11127	3000.5298
A_24_P231829	BHMT2	16.252262	17.593092	33.894016
A_33_P3503284	BOD1P	6.8263745	5.981133	12.527021
A_23_P254797	BPIFB2	90.760704	83.15059	237.89432
A_23_P89973	BRSK1	32.37556	26.66769	91.67442
A_33_P3288431	C12orf50	10.746909	17.05764	35.182755
A_33_P3403459	C14orf167	512.9974	441.87592	981.4865
A_33_P3396275	C15orf62	194.47903	140.84279	357.31705
A_24_P456490	C1orf204	8.098513	15.395879	25.641928
A_23_P3956	C1QTNF1	349.87204	345.52832	1020.1469
A_33_P3380772	C20orf160	3756.2148	3655.0544	11135.831
A_23_P132139	C21orf58	347.93884	290.62076	1085.799

A_33_P3394252	C2orf67	34.688705	27.868618	55.686687
A_33_P3347869	C3	410.73798	502.32687	1140.1812
A_23_P2431	C3AR1	6571.2256	7387.1514	15021.205
A_23_P312383	C5orf64	7.9948153	8.896511	14.6351
A_24_P100650	C9orf11	4.9999995	5.2086687	6.9475245
A_24_P401870	C9orf139	15.436991	10.972792	28.59563
A_33_P3359223	C9orf173	1308.318	1155.6991	2545.752
A_23_P307502	C9orf53	216.41455	189.42609	1053.3795
A_24_P303874	C9orf62	815.2112	502.31094	2717.0735
A_33_P3334398	CA6	635.76227	648.6666	1358.1013
A_33_P3318646	CALY	7464.1274	7242.137	22848.09
A_24_P292929	CAMKK1	10.118651	12.004202	23.50445
A_23_P138835	CAPN1	1877.1244	2085.2048	5174.0835
A_23_P217570	CAPN6	7.224693	10.600702	16.244148
A_23_P152406	CAPNS2	784.1337	653.3245	1506.6105
A_23_P126752	CAPZB	5667.146	5453.0312	12272.863
A_24_P3045	CASP10	12.08541	15.1755705	23.889343
A_33_P3257594	CCDC103	19.160599	13.386822	34.54912
A_33_P3261054	CCDC114	11.218641	22.625563	39.943283
A_33_P3246613	CCDC78	6842.084	7136.498	16831.371
A_23_P207456	CCL8	6.340178	19.452358	33.151234
A_23_P343398	CCR7	26.99001	31.058153	41.94847
A_33_P3234292	CD244	23.800396	22.742418	41.29029
A_33_P3245439	CD40	10.8409	16.876284	33.906578
A_23_P40192	CDH22	2244.1453	2382.2957	5369.2783
A_23_P154050	CDK15	36.533554	33.27653	292.59503
A_24_P118376	CEACAM20	4669.6055	3786.65	10017.041
A_33_P3671378	CERCAM	383.16586	312.1826	805.84625
A_23_P5679	CFC1	4.9999995	8.237859	11.970499
A_33_P3376965	CHAC1	2152.0273	1435.451	4717.5127
A_23_P349463	CHP2	7.8933153	9.4271	15.345508
A_33_P3345186	CHST13	110.01203	76.172874	216.62065
A_33_P3299739	CLDN19	1793.792	1176.0189	3568.879
A_33_P3775848	CLIC2	5.8539605	8.739239	14.199943
A_23_P215479	CLIP2	1139.3367	930.72015	2098.9053
A_24_P269551	CLVS1	4.9999995	4.9999995	10.914868
A_23_P37718	CNGB1	56.267166	39.023045	96.37552
A_33_P3220347	CNPY1	30.522774	26.130253	49.027847
A_33_P3216448	COL11A2	202.21225	215.1566	785.67316
A_33_P3269539	COL6A2	5323.8877	5579.7944	15041.038
A_33_P3210488	COL6A3	8308.847	6329.138	15648.159
A_23_P2474	COPS7A	1487.7974	1373.6381	2840.3655
A_33_P3378284	COX6B2	3868.9683	2975.1443	9286.767
A_33_P3343196	CP	6.6754756	5.836154	18.808216

A_33_P3222424	CSF3	1371.932	314.37628	2423.5107
A_23_P170453	CST5	775.81476	723.8127	1700.8323
A_33_P3357979	CTBP1	335.72968	316.92584	755.501
A_32_P86150	CTRB2	1457.7062	1178.489	3076.1238
A_33_P3355580	CTSL3	20.808908	17.952648	296.6196
A_33_P3712341	CXCL12	333.33563	291.34283	521.9166
A_33_P3261132	CYCSP52	53.63392	30.849384	118.466606
A_33_P3285868	CYGB	2332.327	1731.1384	5620.17
A_23_P215997	CYP11B2	6.2548456	10.37883	16.32613
A_23_P208373	CYP2B6	123.29476	106.20361	346.0602
A_33_P3303474	CYP4A11	17.328028	11.14476	27.112778
A_23_P146198	CYP7A1	4.9999995	8.6325655	11.785751
A_23_P115161	DARC	5.345743	8.400822	11.821681
A_23_P212105	DAZL	12.177572	13.180187	24.480429
A_23_P50399	DCAF15	184.45161	103.592285	392.43973
A_33_P3768992	DCAF4L2	5.226402	35.004555	85.459
A_33_P3346083	DCDC1	7.397402	11.853419	32.4891
A_33_P3264577	DCTN1	2288.6152	2004.3037	4507.34
A_23_P93311	DDR1	379.52832	568.3353	747.24023
A_33_P3724155	DERL3	1200.8143	933.4657	3122.8887
A_33_P3330125	DIABLO	3109.8345	3258.4724	6955.773
A_24_P236251	DLK1	22.837652	18.52143	42.196243
A_23_P359457	DNAH2	99.43101	79.95397	188.62851
A_23_P255876	DNAI1	10.05623	11.767361	28.286093
A_33_P3285629	DNAI2	7.440089	9.154515	13.9415
A_24_P50091	DNM1P46	23.73833	24.388523	43.50886
A_24_P359405	DTX3	60.98432	59.700737	119.94491
A_23_P347432	DVL1	446.7325	319.22818	819.4416
A_23_P171132	EDA2R	28.928463	22.62024	75.50574
A_32_P83049	EFR3B	888.5846	887.48865	2168.9597
A_23_P20894	EHMT1	153.00508	131.25917	285.66022
A_33_P3402116	EIF2C1	1908.3202	1505.8757	3985.8862
A_33_P3268478	ENO4	4.9999995	12.885479	19.461363
A_23_P351	EPB41	13.601318	19.226429	32.341446
A_24_P63537	ERAP1	4.9999995	7.1176586	10.30415
A_24_P314585	ERAS	24.75101	26.317026	57.728813
A_33_P3235432	ESYT3	238.95398	218.05273	662.5407
A_23_P8820	FABP4	9.245049	13.43238	26.16549
A_33_P3424861	FAM118A	1041.5835	970.2659	3412.0127
A_33_P3279124	FAM21C	3599.1353	2845.782	7034.3105
A_24_P941898	FAM22D	28.064476	23.07835	43.95513
A_23_P45488	FAM3A	278.0447	309.96448	680.29156
A_32_P70203	FAM41C	8.87177	13.453199	25.558752
A_23_P21560	FAM49A	26.225498	31.623318	63.61314

A_23_P364444	FAM65C	17.32068	8.565667	28.717138
A_32_P6221	FAM86C2P	38.568775	39.663002	85.20486
A_33_P3257513	FAT3	1244.7169	921.11993	2725.9192
A_23_P200637	FBXO44	92.87148	86.60386	241.66374
A_23_P160849	FCER1G	739.4592	645.2411	1353.8069
A_24_P276576	FCRLA	6.772625	10.02719	20.165169
A_33_P3380797	FGF3	36.10452	27.773647	70.079445
A_23_P316447	FGFR1	22.48515	19.525677	40.070698
A_24_P147540	FLJ22184	5640.107	4369.0483	17799.59
A_33_P3739870	FLJ31183	26.138071	8.483534	54.025085
A_33_P3325843	FLJ40039	419.46707	355.61554	1111.001
A_33_P3421084	FLJ42289	6.7124248	8.458752	11.590111
A_24_P252996	FOLR3	8.877824	11.708019	18.41986
A_33_P3264331	FOXD3	5.0444684	6.34313	9.387477
A_33_P3269403	FOXI1	4.9999995	4.9999995	10.23001
A_33_P3249793	FOXO4	10.272988	12.979392	47276.707
A_33_P3284019	FOXP4	3569.4924	2918.8525	13327.459
A_24_P738168	FREM3	4.9999995	5.218929	6.9840865
A_32_P160896	FTMT	222.89832	174.24857	643.5457
A_24_P393740	FYB	5.612754	4.9999995	17.933393
A_23_P252462	GALNT9	69.66866	45.429695	140.73468
A_33_P3323822	GATAD2B	36.991116	34.954185	77.28077
A_23_P130642	GBP6	2018.7867	1626.5309	3773.9456
A_32_P197621	GEMIN8P4	4.9999995	4.9999995	11.29536
A_33_P3377100	GHRH	22.98347	16.921608	50.116623
A_33_P3273767	GJB7	4.9999995	4.9999995	23.264036
A_33_P3264790	GJD4	17.29674	18.477417	30.238426
A_33_P3285715	GLI4	6679.593	6092.8467	17635.752
A_23_P67913	GMPPA	356.6344	340.1793	752.49054
A_24_P319374	GPA33	4.9999995	4.9999995	6.577977
A_23_P416686	GPR137	166.3395	167.98088	380.02615
A_33_P3302586	GPR153	38.81	31.637514	71.5682
A_23_P142096	GPR32	113.4688	101.21466	423.48154
A_33_P3369371	GPX3	8.7216835	11.86791	27.992579
A_32_P380675	GRIP2	8.769227	7.167501	26.132267
A_23_P58869	GSTM2P1	100.991066	104.038315	253.492
A_23_P41304	GYPE	8.134949	11.92808	19.527039
A_23_P28697	HAAO	236.46577	184.64052	506.16745
A_33_P3319640	HDGFRP2	219.68857	165.88336	459.1838
A_23_P149221	HECTD3	204.83034	185.30913	405.3724
A_24_P213950	HEPACAM	21.585857	25.094572	43.366066
A_24_P51201	HERC3	13.438958	12.767268	25.85841
A_23_P250358	HERC6	4.9999995	9.303241	15.611818
A_33_P3322859	HES6	2110.1333	2018.0422	4911.6807

A_33_P3338152	HIF3A	2066.5056	1635.9553	4688.3984
A_23_P40347	HM13	2015.8291	1705.0525	3789.189
A_33_P3598466	HSP90AB6P	187.85297	104.694405	404.59015
A_33_P3331120	HSPBAP1	4.9999995	9.592722	13.867079
A_33_P3212665	HTATSF1P2	96.94246	102.5124	234.17934
A_33_P3421984	HYAL4	1735.94	1566.4999	3500.5132
A_33_P3423551	IER3	4740.129	2456.847	5840.357
A_23_P13907	IGF1	22.903273	19.563646	90486.32
A_24_P382187	IGFBP4	5180.8223	6726.9834	15495.363
A_33_P3371954	IGSF8	104.29571	103.85911	223.25415
A_23_P166775	IL17RC	238.94522	288.94693	776.5726
A_33_P3374190	INPP5A	124.050064	101.09239	242.25346
A_32_P178800	ITGA2	1176.8625	790.4658	1733.2545
A_23_P36562	ITGA5	5723.1455	4755.7305	13252.723
A_33_P3398143	ITGB2	158.05054	114.024536	171.76271
A_33_P3806721	JMJD5	635.5576	525.6821	1190.8912
A_33_P3220929	JOSD2	71.460205	56.79389	179.50595
A_33_P3326020	KANK3	166.30942	151.79167	405.16586
A_33_P3395823	KCNQ2	438.43796	370.7622	948.38306
A_33_P3290955	KIAA1875	1881.5011	1615.3722	4920.1133
A_33_P3330643	KIF6	4.9999995	8.924842	56.879498
A_24_P376047	KLK4	9.798246	14.224777	22.724497
A_33_P3315263	KRT79	17.098412	22.570015	68.39792
A_33_P3273534	KRT81	12266.407	10800.171	31019.543
A_23_P15734	KRT9	193.9567	152.42554	366.80035
A_24_P142495	KRTAP1-3	3103.056	2555.4248	6288.71
A_33_P3251421	KRTAP21-1	144.64203	100.943184	266.32657
A_33_P3388061	KRTAP21-2	8.7592745	10.397809	18.832228
A_23_P153022	KRTAP2-4	3821.783	2496.7808	7256.2593
A_33_P3363310	KRTAP5-10	461.7293	346.92096	876.62787
A_24_P11506	KYNU	91.32153	71.71939	173.06717
A_33_P3277532	LAMC3	9.814722	13.882019	24.89652
A_23_P86493	LBX1	6676.11	6143.6562	15129.918
A_23_P63521	LCE2C	7.8203964	15.52767	67.13546
A_23_P369471	LCE3A	26.041122	16.416971	105.77049
A_33_P3423931	LCN9	141.38783	105.139626	216.00266
A_33_P3236441	LDHAL6A	1351.7725	1176.8013	3163.6216
A_33_P3405022	LETM1	253.30156	211.2666	618.6198
A_23_P434518	LFNG	347.78708	291.06485	788.4545
A_33_P3310274	LGR6	14.70221	13.271608	22.563162
A_33_P3223678	LHX3	1620.0239	1466.2378	4690.612
A_33_P3268181	LIMS2	103.2851	96.33829	239.82877
A_33_P3403082	LINC00176	2165.925	2031.4722	6091.2886
A_33_P3278813	LOC100127885	5.5601974	12.521021	23.320822

A_33_P3293469	LOC100128105	9.291117	8.741138	14.466649
A_33_P3397180	LOC100129324	376.4139	317.59338	875.1867
A_33_P3364964	LOC100129596	415.7665	274.01447	837.4492
A_33_P3287661	LOC100130430	248.21709	255.2769	898.8748
A_33_P3314974	LOC100130522	492.23355	300.62598	934.6092
A_33_P3351259	LOC100130560	265.36392	264.36664	649.66907
A_33_P3298092	LOC100131756	18.050398	12.94697	33.25587
A_33_P3386686	LOC100132874	2408.4075	1753.9547	4376.917
A_33_P3236340	LOC100133131	478.4627	412.8514	1104.5349
A_32_P45974	LOC100134167	18.041016	22.36186	43.3951
A_33_P3875565	LOC100270679	50.104927	38.72071	97.346405
A_33_P3518572	LOC100287314	604.6346	437.1172	1164.3741
A_33_P3233459	LOC100287879	140.48401	121.14562	402.60495
A_33_P3419621	LOC100289580	64.4412	58.378815	118.50413
A_33_P3233070	LOC100505564	26.840431	29.653402	62.72697
A_33_P3392000	LOC100506190	1455.3713	1177.1599	3367.719
A_23_P436145	LOC100507431	4.9999995	4.9999995	9.922209
A_33_P3415247	LOC115110	7.301587	5.439678	18.28068
A_33_P3452003	LOC143286	504.6033	423.10687	996.3411
A_33_P3339436	LOC145845	87.12869	84.04632	293.9754
A_24_P520767	LOC149351	19.44016	21.669342	35.693394
A_24_P918907	LOC154761	10.010069	14.41083	26.045614
A_33_P3464749	LOC253044	4.9999995	9.488324	13.688929
A_33_P3870056	LOC283335	147.20842	109.88357	534.8474
A_33_P3336587	LOC283710	66.05248	44.79729	115.01159
A_33_P3663105	LOC284263	4.9999995	11.275981	20.06314
A_33_P3317623	LOC284379	480.3613	514.0612	1356.8881
A_33_P3461633	LOC284454	356.3099	309.59464	879.50275
A_24_P33446	LOC338651	4.9999995	6.84043	13.645407
A_33_P3832872	LOC389247	5.408224	5.489724	11.16878
A_32_P202703	LOC389831	821.559	540.76514	15780.206
A_33_P3550618	LOC400541	21.333595	16.067886	32.937527
A_33_P3235132	LOC400968	1428.3899	1069.1561	2656.4155
A_33_P3780572	LOC401164	16.685528	16.827765	30.25871
A_33_P3344690	LOC442421	4.9999995	4.9999995	32.142647
A_24_P144303	LOC442459	187.40144	150.89633	436.9219
A_33_P3293391	LOC642826	258.36377	233.24072	522.37604
A_32_P60635	LOC643037	4.9999995	6.326512	8.824883
A_33_P3300332	LOC644093	34.475445	38.259983	107.61461
A_33_P3339720	LOC644841	7.156799	4.9999995	16.166918
A_33_P3667484	LOC648740	11004.355	7646.4463	22295.143
A_33_P3395442	LOC678655	68.47123	81.8008	175.84273
A_32_P173114	LOC729444	4.9999995	5.0886765	7.656175
A_23_P436048	LPAR3	31.85344	25.922873	51.589188

A_23_P153461	LPPR2	200.4694	235.62444	539.5444
A_23_P163195	LRFN5	4.9999995	7.966208	11.421339
A_33_P3391105	LRP1	675.8711	663.0362	922.0008
A_24_P99216	LRP10	1555.6791	1431.055	3348.026
A_24_P827037	LRRC15	29.411993	17.903082	56.272167
A_24_P240259	LRRC31	4.9999995	4.9999995	12.158399
A_33_P3420446	LRRD1	190.13432	138.11653	593.719
A_33_P3418194	LYNX1	621.9147	493.76163	2742.252
A_23_P62099	MAGEC2	4.9999995	4.9999995	6.511168
A_23_P163455	MAP1A	2300.875	2208.2961	5746.5396
A_23_P308483	MAP3K15	8.182247	8.978967	18.410477
A_24_P119201	MBD2	9.934969	7.328211	16.507586
A_23_P50121	MC4R	9.709909	11.1790495	17.874731
A_32_P850175	MCF2L2	10.13384	7.5501876	19.739027
A_23_P28507	MGAT4A	5.608346	6.289719	9.372455
A_32_P183918	MGC39372	16.739468	14.7769	26.089159
A_23_P13873	MLF2	4619.283	3877.0022	8357.145
A_33_P3415216	MPV17	3754.0083	3780.6199	5248.88
A_24_P146683	MSMB	6.9790688	11.560231	17.533607
A_23_P129629	MT3	3537.8735	2226.32	5354.912
A_23_P152125	MVD	184.32596	306.88812	760.6625
A_33_P3255829	MXRA5	4.9999995	6.0080853	13.026199
A_24_P324314	MXRA7	918.6445	748.90765	1726.5294
A_23_P258018	MYL5	412.68198	344.43005	805.7647
A_33_P3238157	MYO18A	221.44519	209.9552	465.35452
A_23_P78122	MYO18A	482.42114	547.09436	1180.8346
A_33_P3395688	MYZAP	7.6978035	7.3183293	18.917063
A_23_P138194	NCF2	4.9999995	4.9999995	8.253653
A_23_P29638	NCKIPSD	118.061066	136.13339	307.2913
A_23_P9614	NDUFA4L2	11.43466	18.114784	34.570194
A_24_P99639	NEK8	5.4288726	12.952899	65.685486
A_33_P3293524	NEURL	573.0149	356.3073	1571.3599
A_33_P3856171	NF1P2	10.563909	8.544691	23.675388
A_33_P3260342	NFASC	16.216602	17.38201	37.00738
A_33_P3225600	NFE2L2	94.21539	56.384766	379.15768
A_23_P202156	NFKB2	319.2624	263.9718	737.6105
A_33_P3386506	NFKBIL1	5105.737	4366.594	11070.546
A_33_P3299599	NKX2-5	4800.8354	4744.3765	15700.566
A_23_P340019	NLRC3	4.9999995	6.0873084	11.949249
A_24_P918384	NSD1	44.65188	40.770264	107.73917
A_23_P44836	NT5DC2	5465.8394	5430.7715	13122.719
A_24_P391586	OAF	571.40546	407.7223	1117.4958
A_23_P64828	OAS1	9.009503	24.848978	43.10678
A_24_P335305	OAS3	77.87376	68.03186	141.57222

A_33_P3379680	OR2A5	24.070017	19.354853	55.591576
A_23_P111092	OR2H1	5155.439	3616.8894	9591.939
A_33_P3360157	OR5AS1	5.3366446	4.9999995	9.228104
A_23_P254852	OTOF	2168.6443	1785.1403	3816.272
A_23_P127721	P2RX3	161.20464	158.90848	428.58392
A_33_P3394933	P4HA2	273.8868	260.19095	566.18854
A_23_P48307	PABPC3	7011.0576	5068.4746	14490.068
A_23_P126869	PADI3	13.9582205	10.191319	22.642784
A_33_P3358213	PADI6	11.24494	11.406299	17.36259
A_33_P3216890	PAG1	4.9999995	6.653037	13.08615
A_33_P3588134	PANX2	51.97705	46.548206	103.464584
A_23_P40718	PARVB	618.53064	539.2463	1352.0219
A_33_P3224362	PCBP3	28.766157	21.006865	62.212772
A_33_P3262927	PCBP4	254.24626	212.70805	491.51984
A_33_P3332521	PCDHA3	8.4072695	9.09601	13.172782
A_24_P14731	PCSK1N	1874.0273	1809.8511	4543.056
A_23_P23090	PDC	10.03621	11.0642	19.054798
A_23_P208334	PDE4A	51.18814	48.34205	92.2323
A_23_P149992	PDLIM1	972.15155	929.3897	1954.0232
A_33_P3217213	PDLIM7	2411.047	2012.6615	4722.8516
A_24_P339944	PDGFB	139.495893	128.487234	198.73865
A_23_P29079	PFKL	511.7114	501.6675	3167.1765
A_23_P350059	PFN1P2	9617.157	6908.0493	28877.848
A_23_P80684	PHF7	20.34176	18.10197	77.43269
A_23_P1519	PITPNM1	195.86589	194.17113	411.45758
A_23_P67271	PKN1	779.5603	731.8019	1584.073
A_23_P88222	PLD4	7.898032	15.3332615	31.178026
A_33_P3272539	PLEKHG5	1974.2712	1592.0123	3714.4072
A_23_P5983	PLTP	600.4754	661.3265	1512.262
A_33_P3321034	PLXNA4	229.1182	187.20372	461.04977
A_23_P10077	PNPLA2	287.72745	291.02023	687.9377
A_23_P16166	PNPLA6	770.33246	727.3601	2033.1124
A_23_P211227	POFUT2	156.39061	188.00117	541.39966
A_23_P215669	POLR2J2	295.4269	264.5175	802.222
A_23_P252062	PPARG	67.1474	119.04879	180.25668
A_33_P3239123	PPIH	27.955992	25.280125	43.81241
A_33_P3594854	PPIP5K1	15.994747	13.113091	28.012339
A_33_P3252048	PPP1R27	279.70087	243.07716	818.00415
A_23_P60458	PPP2R4	2160.2427	2059.0278	4531.2095
A_23_P119448	PPP6R1	78.05328	62.446056	182.2467
A_24_P396197	PRKCSH	1070.4382	910.31024	2162.4644
A_24_P349466	PRR13	437.68536	251.21716	1147.1273
A_33_P3222769	PRR25	1614.801	1553.2941	3658.7117
A_24_P412734	PRSS36	398.17596	341.33438	890.8554

A_33_P3329088	PRSS8	21.412249	15.3079405	132.98608
A_23_P68121	PSD4	33.709854	30.42616	57.616863
A_23_P325080	PTOV1	660.4667	689.0477	1600.7446
A_23_P396062	RAB40C	251.93425	262.0271	665.1328
A_23_P54223	RABGGTA	176.08432	179.10118	387.03064
A_24_P228717	RAC2	832.6898	673.28564	1619.2146
A_33_P3276323	RAD21-AS1	4.9999995	6.896128	11.185961
A_23_P151307	RAPGEF3	14.521219	23.660275	39.771145
A_33_P3499102	RBM10	444.57962	317.16916	932.88336
A_23_P67339	RCN3	2331.2695	2045.0522	4686.924
A_33_P3259861	REREP3	759.58167	570.29266	1428.9933
A_33_P3222962	RIBC1	4.9999995	8.73123	23.846466
A_33_P3336632	RN28S1	111.95033	410.0828	1045.7554
A_24_P297480	RNF113B	29.20837	26.840555	64.008545
A_24_P344295	RNF167	166.95015	130.32417	322.89682
A_23_P364890	RNF40	431.39618	350.65604	813.73004
A_32_P203013	RPS10P7	10708.734	8521.9375	29480.885
A_24_P242036	RRP7B	828.81006	678.8976	1631.8995
A_24_P55225	RSPH9	198.88449	211.04146	581.48627
A_24_P221575	RUFY3	115.64557	98.43985	220.13405
A_33_P3409086	S100A1	57.88141	39.910976	106.669525
A_23_P435941	SAMD1	557.0909	442.3646	1036.479
A_33_P3220152	SBNO2	468.01624	379.80966	927.93115
A_24_P117672	SCAF1	863.194	684.4654	2243.5415
A_23_P105144	SCUBE2	10.176085	18.00499	31.006561
A_33_P3287529	SDF4	2759.85	2861.255	6153.3174
A_33_P3348224	SEC14L6	8.01586	14.004959	35.06084
A_33_P3474175	SFTA1P	2669.274	2351.1113	8465.863
A_23_P130553	SHKBP1	479.81418	599.57623	1491.2563
A_24_P304311	SLC22A8	11.9052	17.370478	30.027246
A_24_P111054	SLC2A5	538.9491	424.42877	1091.1118
A_33_P3385006	SLC39A5	20.989243	13.007971	36.529305
A_33_P3241696	SLC45A2	13.65756	39.384727	91.57979
A_33_P3411035	SLIT1	8.811284	8.850543	193.9951
A_24_P83922	SNRPC	155.47775	87.23574	448.7576
A_33_P3414192	SORCS1	35.995426	28.294556	69.411446
A_33_P3325110	SOX13	448.6001	389.19107	881.6215
A_33_P3233608	SPANXB2	5.7937512	9.162595	20.570704
A_33_P3216438	SPATA21	68.58515	46.723324	142.56274
A_23_P258381	SPSB4	7088.607	5122.691	18305.896
A_33_P3236798	SRMS	203.76521	183.10425	458.42224
A_23_P54968	ST6GALNAC1	20.01962	16.036753	30.775753
A_33_P3366758	ST8SIA6	8.30851	14.935391	133.44669
A_32_P92783	STIP1	1799.5652	1206.5153	3238.0073

A_33_P3335920	SYNE1	734.4067	635.03986	1825.973
A_23_P20101	TAS2R4	78.99555	64.83029	151.77983
A_33_P3219197	TBC1D10B	88.29411	54.685974	155.90846
A_33_P3307886	TBXA2R	525.3497	450.39688	1360.1605
A_33_P3400057	TCEANC	15.81698	13.364951	24.074234
A_33_P3261695	TEX22	4.9999995	11.229329	16.845488
A_24_P79054	TGFB1	1660.6471	1501.2599	2823.036
A_32_P86763	TGM2	1199.1533	1689.2063	3769.4302
A_24_P196851	TLN1	3962.7017	3104.72	8767.292
A_23_P256561	TLR6	6.0252657	9.67972	20.304895
A_24_P932416	TMEM14E	5.398946	10.101289	15.207691
A_23_P112289	TMOD1	8.09312	9.6086035	15.966283
A_23_P418597	TNKS1BP1	604.78485	490.69168	1533.2938
A_23_P34700	TNNT2	37.676346	21.81879	69.17029
A_23_P26294	TPSG1	12443.622	10055.234	37141.79
A_33_P3357600	TRIM3	167.43456	115.218796	369.06058
A_23_P123402	TRIM55	21.853016	18.675753	47.445965
A_24_P392632	TRPM3	9.763314	9.6524105	15.714332
A_24_P200942	TSC22D4	446.6974	422.8737	908.562
A_23_P76136	TSPAN11	4.9999995	4.9999995	7.323604
A_23_P114185	TSPAN7	10.356913	10.031314	16.017202
A_33_P3234222	TSPO2	2403.2278	2315.1277	5902.6353
A_23_P170608	TSPYL2	90.72399	72.49553	164.7834
A_33_P3544887	TTC28	265.05658	230.65193	635.3518
A_33_P3409518	TUBBP5	32.52201	45.8622	100.00402
A_23_P308058	TUSC5	20.289358	20.388311	54.205467
A_23_P53015	TUT1	18.905668	33.190403	75.28751
A_23_P141917	TYK2	68.28769	77.65811	177.34866
A_33_P3255949	TYMP	11821.123	14373.168	36537.45
A_33_P3239214	TYR	202.63107	231.852	2538.313
A_23_P137209	UBA1	4596.855	3701.9133	10824.443
A_33_P3344599	UBE4B	7.7075233	7.374841	17.79714
A_23_P120822	UPB1	392.45737	362.31424	901.00635
A_23_P64954	USP5	413.17468	418.6023	961.9376
A_23_P146885	UTS2R	1655.8502	1478.0673	3288.8918
A_33_P3294886	VPS52	11.552419	18.901308	57.127254
A_33_P3316493	WDR91	44.512543	53.643406	105.5494
A_23_P402331	WFDC5	43.949158	37.79562	70.805435
A_33_P3605352	WWTR1	22.889877	21.294258	37.721638
A_23_P153628	YIF1B	1372.176	1316.8375	2726.75
A_23_P104804	ZBTB16	138.53046	130.69246	309.56006
A_32_P65533	ZC3H12D	4.9999995	6.001008	13.19199
A_24_P99175	ZDHHC22	6.2782927	6.385169	13.975491
A_23_P39223	ZNF2	290.19955	247.07362	697.7215

A_24_P56689	ZNF205	506.43854	461.93478	1065.7261
A_24_P218074	ZNF467	1419.6825	1320.2987	3277.526
A_33_P3239143	ZNF497	11678.706	8756.658	25811.842
A_33_P3228637	ZNF584	11.08674	9.961448	19.302372
A_24_P247978	ZNF589	50.90116	43.46304	89.39675
A_23_P74114	ZNF713	1203.7994	977.00256	2826.0364
A_24_P161144	ZNF843	164.85068	122.135414	340.09024
A_33_P3412294	ZSCAN2	267.54147	222.26337	522.04193

Genes down-regulated in UC-MSCs-IGF-1

ProbeName	GeneSymbol	UC-MSCs	UC-MSCs-vector	UC-MSCs-IGF-1
A_23_P202520	ABLIM1	514.0748	550.9202	195.34111
A_33_P3395008	ACOXL	100.17611	62.07325	21.440973
A_23_P95536	ADAM29	16.663626	16.53795	4.9999995
A_23_P94296	ADAM7	105.615425	80.52821	33.23314
A_23_P81158	ADH1C	648.36523	452.6922	218.26738
A_33_P3348079	ADIG	44.0041	30.31078	11.523061
A_33_P3378081	AGBL1	11.573048	13.16991	4.9999995
A_23_P136116	AGMO	22.181915	14.45243	4.9999995
A_33_P3346771	AKD1	13.929191	13.80182	4.9999995
A_23_P96478	AMELX	34.073597	19.40487	4.9999995
A_33_P3404676	AMOT	78.64373	43.8522	9.283251
A_23_P131875	ANKRD60	82.811295	68.38661	14.157619
A_24_P929369	AP4E1	1571.0555	1375.148	707.79364
A_23_P142856	AQP12A	179.02951	153.758	52.823093
A_32_P83784	ARAP2	11.85679	15.67101	4.9999995
A_23_P35045	ARHGAP30	283.59625	154.6592	53.815556
A_23_P32308	ATP4B	18.836983	19.44749	5.501481
A_33_P3415191	ATP8B1	9173.64	7127.05	3841.7576
A_33_P3286958	B3GALT2	250.15114	460.6793	100.75299
A_23_P19723	BMP5	53.469894	26.39618	8.108629
A_33_P3385453	BRCC3	19.108652	22.94648	5.973767
A_33_P3320743	BSPH1	27.118647	26.67607	8.922841
A_23_P75179	C10orf92	1240.9016	1174.266	511.86194
A_32_P886589	C12orf61	13.078512	12.37969	4.9999995
A_33_P3352718	C12orf76	15.620229	12.15496	4.9999995
A_32_P183970	C15orf62	3723.3975	3430.773	1505.0765
A_32_P468341	C1orf127	350.78726	342.4083	146.11557
A_32_P42946	C1orf210	46.683884	30.36138	10.330431
A_33_P3352727	C20orf173	48.14046	26.16556	4.9999995
A_23_P132079	C21orf62	35.45157	21.33427	7.125975
A_24_P272313	C2orf55	33.39639	46.10329	6.9543223
A_23_P324419	C2orf57	20.744371	12.07015	4.9999995
A_33_P3337569	C4orf40	148.10768	74.35826	27.125711
A_33_P3416966	C6orf168	21.06462	18.00538	5.4825826
A_32_P74964	C6orf168	24.83979	23.27697	5.908457
A_23_P253350	C8orf4	10.88411	19.14648	4.9999995
A_33_P3482249	C8orf66	53.941708	59.97786	16.934801
A_32_P203917	C9	34.596096	18.8335	4.9999995
A_33_P3225705	C9orf102	16.060947	15.36464	4.9999995
A_33_P3280106	CACNA1B	66.94227	62.28838	24.85878
A_33_P3421913	CADM1	2554.8218	2474.52	881.50616
A_33_P3217804	CAMSAP1	3194.665	2606.334	632.3238

A_23_P13102	CASP12	22.708399	30.79887	4.9999995
A_23_P342108	CCDC116	47.479816	54.88309	11.943901
A_33_P3246623	CCDC18	141.96928	91.49139	33.02694
A_23_P105664	CCDC59	16822.473	13754.41	5004.4663
A_24_P125335	CCL13	52.43446	28.64262	10.322151
A_33_P3354607	CCL4	13.937498	14.39675	4.9999995
A_23_P402670	CD1A	35.834248	17.63198	4.9999995
A_32_P204239	CDHR3	18.854319	16.58399	4.9999995
A_33_P3289721	CDRT1	144.71342	125.9066	50.43082
A_33_P3413895	CDRT15P1	24.068768	19.57924	4.9999995
A_23_P153301	CEACAM5	33.062576	13.9821	4.9999995
A_23_P66967	CETN1	24.096022	24.08418	6.41636
A_32_P209960	CIITA	48.46485	37.62087	13.32299
A_33_P3295328	CLEC6A	16.55107	17.73318	5.069837
A_33_P3396404	CLLU1OS	123.80971	86.32254	33.153347
A_23_P371861	CNBD1	12.84181	17.19678	4.9999995
A_23_P216361	COL14A1	46.854202	34.67941	11.256869
A_23_P112554	COL15A1	24.669209	25.21375	7.5115547
A_23_P31124	COL21A1	71.69782	42.90347	10.90868
A_33_P3372069	COX8C	11.94688	10.83257	4.9999995
A_23_P327551	CPNE4	14.515491	11.41224	4.9999995
A_23_P394986	CREG2	442.75748	371.0785	147.81075
A_24_P342484	CRP	28.71284	17.69104	4.9999995
A_32_P81879	CST9	28.233414	22.61938	6.8371806
A_24_P396167	CTSW	12.409269	11.94057	4.9999995
A_23_P18452	CXCL9	20.01243	24.24159	4.9999995
A_32_P410753	CXorf59	10.14364	10.7043	4.9999995
A_23_P37410	CYP19A1	60.446075	53.16777	18.120935
A_33_P3264662	CYP27C1	27.410597	20.39176	4.9999995
A_23_P55779	CYP2A13	4198.552	3566.766	1253.2704
A_23_P12767	CYP2C9	22.610731	12.94495	4.9999995
A_23_P90626	CYTIP	14.4615	16.28876	4.9999995
A_23_P500328	DCX	707.2806	489.3859	251.99646
A_23_P2317	DDN	232.84505	200.4189	85.75207
A_23_P31816	DEFA3	113.2476	66.52535	27.69918
A_24_P363711	DEFA6	15.117859	14.16349	4.9999995
A_33_P3370454	DEFB108B	35.96164	17.52236	5.6165915
A_33_P3421728	DGKG	1137.9961	639.9171	297.46606
A_23_P317450	DNAJB3	18.613592	15.90101	4.9999995
A_23_P401691	DPYSL5	30.518335	18.25582	5.22925
A_33_P3301932	DSCR4	12.48321	14.09461	4.9999995
A_33_P3254956	DSCR6	66.90785	41.10794	14.14076
A_23_P158257	DUX3	60.711193	55.4295	21.368027
A_33_P3373165	DUX4L4	168.29036	154.9886	49.182945

A_23_P404536	ENPP3	284.4757	210.0615	79.88677
A_33_P3280471	ERBB3	19.647861	13.83876	4.9999995
A_32_P183765	ERBB4	48.39991	32.14484	10.013261
A_33_P3226425	ERP29	798.0567	968.5512	299.28757
A_32_P901770	ESPN	141.0072	172.4498	53.07403
A_24_P697685	ESYT3	15.375652	10.86795	4.9999995
A_23_P424734	EXOC3L1	297.72653	226.2664	95.48217
A_33_P3216763	EYA1	12.840409	13.37575	4.9999995
A_23_P84860	FAM107A	14.98117	19.41574	4.9999995
A_23_P36888	FAM113B	1925.8895	1618.933	777.53827
A_33_P3508342	FAM135B	22.486017	16.83151	5.25487
A_24_P561341	FAM150B	15.396858	11.74393	4.9999995
A_33_P3320197	FAM150B	11.288481	12.72261	4.9999995
A_23_P22647	FAM155B	102.4236	90.07399	26.246326
A_32_P84242	FAM169A	46.848396	32.57773	11.723979
A_24_P263310	FAM169B	76.4694	47.44173	12.673541
A_32_P129120	FAM182A	21.415108	19.25762	6.082621
A_23_P422831	FAM189A2	51.676426	40.615	10.13319
A_23_P377629	FAM75C2	134.07819	95.89803	31.900503
A_33_P3368334	FCRL3	56.17008	57.97151	17.749258
A_23_P144126	FETUB	38.252964	29.00265	10.52074
A_33_P3337574	FGF14-IT1	49.774315	40.1952	12.30613
A_33_P3663142	FLJ12334	454.124	244.1552	108.28223
A_33_P3406552	FLJ32955	39.311077	20.69569	6.5864835
A_33_P3331426	FLJ37786	317.76285	277.3294	113.84209
A_33_P3675395	FLJ39095	23.899656	24.82322	7.3190804
A_33_P3294861	FLJ40712	25.77897	27.85171	8.261998
A_33_P3210556	FLJ42289	38.924923	17.20093	4.9999995
A_33_P3354635	FLJ43879	16.09326	12.53404	4.9999995
A_33_P3288219	FLJ45684	242.56128	137.6907	60.827507
A_33_P3263851	FLJ46020	3529.2659	2613.243	1309.8306
A_23_P66552	FNDC8	21.493328	16.96923	4.9999995
A_33_P3355508	FOXL2	16.021591	10.9788	4.9999995
A_23_P55649	FPR2	21.11046	29.13462	5.9313354
A_33_P3248203	FUT5	14.392161	15.93807	4.9999995
A_23_P76749	GALNTL1	147.15695	110.6729	41.629017
A_24_P82466	GAS7	9927.114	12170.23	3875.9695
A_33_P3406962	GATA4	54.173443	40.41768	14.00305
A_32_P140489	GDF6	21.515192	25.281	6.5799265
A_24_P606538	GGNBP1	94.00867	48.65278	17.212843
A_23_P2745	GJB6	17.527824	19.01719	4.9999995
A_24_P43723	GJC3	58.02365	35.90463	14.014919
A_33_P3376273	GK	213.06241	168.7871	79.51718
A_32_P19294	GLT1D1	37.350925	30.9617	9.050743

A_24_P331128	GNA15	295.84024	222.7785	97.42298
A_23_P312901	GPR112	11.230521	15.87674	4.9999995
A_23_P25566	GPR183	149.4899	178.6675	49.471565
A_23_P347541	GRIN3A	12.171659	11.01203	4.9999995
A_33_P3226910	GRK7	16.31181	13.53295	4.9999995
A_23_P76774	GSC	49.406937	43.95129	14.5534
A_23_P92453	GSX2	66.43166	36.46601	7.637982
A_23_P14255	GUCY1B2	52.990948	48.25369	16.813559
A_23_P203558	HBB	54.402218	21.82186	6.3798985
A_23_P64721	HCAR3	52.20944	34.19457	11.74417
A_33_P3360249	HCRTR1	32.333664	17.30232	4.9999995
A_33_P3299872	HINT3	1309.9293	1429.142	551.80804
A_33_P3538688	HIST2H4A	18.867596	19.75426	4.9999995
A_23_P319005	HMP19	12299.852	10375.21	5108.1543
A_33_P3347281	HNMT	19.601368	10.63743	4.9999995
A_33_P3409844	HOOK1	13.87319	10.95047	4.9999995
A_23_P27013	HOXB9	371.79694	316.7939	157.60817
A_23_P118158	HS3ST2	32.850887	31.50624	7.631826
A_23_P422911	HS6ST3	22.026194	21.69409	7.1852193
A_23_P430168	ICA1L	30.019308	22.27966	7.5611844
A_23_P398189	IGSF11	23.096167	23.07895	5.566149
A_23_P7560	IL12B	14.436848	15.49647	4.9999995
A_33_P3211666	IL18R1	12.23331	21.12022	4.9999995
A_23_P28334	IL18RAP	21.8537	18.56892	6.0989447
A_33_P3422124	IL22RA2	11.204881	17.23095	4.9999995
A_24_P230563	IL2RA	51.53198	30.00434	4.9999995
A_23_P125686	INGX	18.941633	14.68626	4.9999995
A_33_P3397995	IPCEF1	29.842121	26.11316	8.926633
A_23_P331598	IPO7	1166.2036	262.6266	116.96431
A_33_P3381707	ISPD	41.12115	30.51226	9.183956
A_33_P3287735	KAT6A	811.3812	526.5867	260.5307
A_32_P75284	KATNAL1	3852.5073	3054.263	1621.823
A_23_P119573	KCNN1	11.1245	12.02532	4.9999995
A_24_P409800	KCTD16	237.61227	121.1253	45.581
A_24_P261750	KERA	20.804548	27.85516	4.9999995
A_33_P3246580	KIAA1244	22.778316	26.28627	6.495582
A_33_P3230548	KIF14	2628.9143	1790.842	770.7751
A_23_P350617	KLB	18.313549	18.07405	4.9999995
A_23_P118854	KRT37	27.480282	18.03886	6.0824504
A_24_P357386	KRTAP13-3	31.221954	20.82705	6.661644
A_33_P3403018	LCE5A	20.961193	25.10687	4.9999995
A_32_P158083	LHFPL3	208.40707	157.0656	50.59803
A_23_P142205	LILRA2	14.839601	11.90579	4.9999995
A_23_P132057	LINC00158	20.564932	16.67414	4.9999995

A_33_P3616021	LINC00163	29.357216	23.88498	8.645869
A_33_P3289960	LINC00222	60.77875	37.91408	13.09992
A_32_P176911	LINC00239	36.598103	27.62146	10.348129
A_33_P3302453	LINC00242	16.064058	10.87003	4.9999995
A_32_P214178	LINC00319	95.21754	92.22371	28.272942
A_33_P3741022	LINC00511	124.88997	111.4446	34.741196
A_23_P258493	LMNB1	2929.8423	672.1179	300.7508
A_33_P3398181	LOC100127909	39.450546	28.85848	10.30083
A_33_P3327041	LOC100128054	297.67676	210.1629	81.959816
A_32_P103614	LOC100128511	154.87404	25.38851	6.576764
A_24_P765552	LOC100128644	106.48681	78.92036	28.24508
A_32_P834166	LOC100128843	9036.325	8665.625	3783.007
A_33_P3213419	LOC100129447	825.1948	735.938	291.40024
A_33_P3382296	LOC100129662	15.897041	11.90882	4.9999995
A_33_P3336552	LOC100130701	17.948608	11.7239	4.9999995
A_33_P3255459	LOC100133461	10.16242	12.58564	4.9999995
A_33_P3805819	LOC100505890	10.39923	14.02682	4.9999995
A_33_P3300867	LOC100506514	103.64619	86.87978	37.662388
A_32_P49867	LOC100507055	35.544422	34.8387	10.662358
A_33_P3289810	LOC100509091	15.89861	12.71861	4.9999995
A_24_P83075	LOC100509196	362.82355	297.131	145.3198
A_33_P3248008	LOC100652807	193.66203	210.3392	73.21898
A_33_P3307945	LOC100652824	486.62808	347.3783	101.17493
A_33_P3738849	LOC147093	37.03069	29.79727	5.138504
A_33_P3482461	LOC150005	33.307583	31.25888	8.074156
A_33_P3289621	LOC283143	60.14919	44.83978	13.737842
A_33_P3274832	LOC283440	62.989933	45.27503	11.61769
A_33_P3337584	LOC283482	25.0315	21.00588	4.9999995
A_33_P3379106	LOC283867	26.153782	26.27409	8.618256
A_32_P232808	LOC284412	96.380005	70.46906	30.260342
A_33_P3463355	LOC286121	12.224959	10.76087	4.9999995
A_33_P3705339	LOC339685	32.32661	21.55776	6.1681275
A_33_P3323979	LOC339874	176.5723	146.8305	56.854424
A_33_P3365553	LOC389634	12.774891	10.72677	4.9999995
A_24_P101642	LOC401847	229.54236	216.1825	72.51122
A_33_P3573141	LOC440149	13.10192	17.24369	4.9999995
A_33_P3688869	LOC440910	110.35122	84.64952	34.27089
A_33_P3770642	LOC503519	57.91553	20.03326	6.0183635
A_32_P102519	LOC541473	50.521328	37.41354	11.608581
A_33_P3336597	LOC554223	240.00893	191.0269	85.8805
A_24_P933685	LOC643797	15.844651	17.10371	4.9999995
A_33_P3306659	LOC728254	3989.9563	3357.148	1540.8523
A_33_P3307536	LOC729444	24.918484	24.61658	8.129662
A_33_P3254375	LOC730651	11.318848	10.65449	4.9999995

A_32_P190303	LONRF2	46.068287	46.10704	14.390751
A_23_P386888	LPA	392.848	260.404	128.67163
A_33_P3292402	LRRIQ3	43.82324	39.91149	11.618931
A_24_P397294	LTC4S	241.47627	198.3876	85.51452
A_33_P3268622	LY6D	31.910036	17.4651	4.9999995
A_23_P153616	MADCAM1	10202.2705	7707.485	3156.8447
A_24_P286898	MAPK8	11055.985	8296.098	2142.428
A_23_P327022	MDFIC	2687.6577	2145.023	1048.2386
A_33_P3315425	METTL4	419.6113	383.0162	176.30986
A_33_P3393331	MFSD6L	14.235211	16.88654	4.9999995
A_24_P912048	MGC50722	20.28246	12.16906	4.9999995
A_33_P3279550	MIXL1	42.182407	30.95339	9.241629
A_23_P13094	MMP10	142.56491	28.88626	8.946831
A_33_P3229477	MPP7	29.977575	34.6485	8.970326
A_23_P4545	MRO	530.2113	317.7589	88.178825
A_33_P3244168	MRPS17	15.86583	17.75992	4.9999995
A_33_P3312499	MS4A15	12.0775	22.42405	4.9999995
A_23_P150979	MUCL1	22.233751	21.65422	6.043578
A_23_P87860	MYF5	75.69389	37.88973	15.031078
A_24_P131646	MYL3	68.80851	63.59367	21.226372
A_33_P3300217	MYLK3	26.467464	21.28867	6.1288366
A_23_P52727	NAV2	4248.1416	1256.782	606.52325
A_33_P3279158	NBPF6	32.419785	30.86493	9.787766
A_33_P3417113	NPSR1	232.56378	115.5601	42.2297
A_33_P3386150	NRADDP	19.172579	13.82629	4.9999995
A_23_P161297	OGDHL	21.171377	45.30272	5.772688
A_23_P154849	OLIG1	25.339636	19.96639	6.7634735
A_33_P3213408	OR10AD1	24.32536	27.30943	4.9999995
A_23_P23507	OR10T2	50.560432	27.76165	8.081972
A_23_P259555	OR12D3	75.39935	66.30253	25.098679
A_23_P169207	OR13C4	52.2132	44.9082	18.056282
A_33_P3244215	OR13C5	15.25674	11.9985	4.9999995
A_33_P3266489	OR13H1	1128.0812	978.0804	421.37244
A_24_P299769	OR1C1	35.811584	27.18985	7.960661
A_33_P3209806	OR2C3	11.69136	11.40818	4.9999995
A_33_P3370001	OR2L8	221.50726	178.2522	70.92869
A_33_P3319834	OR2T8	25.308867	14.76898	4.9999995
A_23_P18913	OR2V2	194.66199	180.6825	58.832462
A_23_P255389	OR2W1	57.459305	39.86321	7.8812
A_23_P13455	OR51A7	931.1509	750.4296	288.28223
A_23_P36203	OR52B2	81.64597	41.3104	14.01744
A_23_P47464	OR5AP2	30.822977	17.39335	4.9999995
A_23_P36050	OR8K3	31.949156	18.01611	4.9999995
A_33_P3262376	OTUD7A	14.744199	12.17283	4.9999995

A_33_P3299540	OVCH2	50.946556	38.64417	10.824549
A_33_P3413741	OXTR	2988.8481	2748.319	923.7499
A_23_P372848	P2RX1	850.7057	791.4865	377.20477
A_33_P3263867	P2RX7	45.14042	31.20255	11.89425
A_23_P346376	P2RY10	621.15704	341.227	109.58301
A_23_P147465	PARK2	430.96835	249.999	113.02656
A_24_P880043	PCGF5	2420.0657	2139.585	921.1035
A_24_P844984	PIGR	45.446304	20.21319	4.9999995
A_23_P66543	PIK3R5	66.824554	58.19374	23.685907
A_24_P294408	PLA1A	17.661917	15.31398	4.9999995
A_33_P3214339	PLCXD2	114.81868	82.42185	34.60361
A_23_P253321	PNOC	12.868958	15.05016	4.9999995
A_23_P147563	PPM1L	28.235607	21.4498	4.9999995
A_32_P38623	PPP1R9A	34.490635	21.69312	6.114865
A_24_P81298	PPP6C	425.82407	343.5278	173.80136
A_33_P3307207	PRHOXNB	241.38586	166.1669	72.362114
A_33_P3821350	PRO2012	67.31281	49.3006	18.608757
A_23_P127663	PRRG4	269.47775	242.963	111.18723
A_33_P3336944	PRSS55	27.40306	27.46449	8.419273
A_33_P3232173	PSPC1	182.79514	66.0746	21.120392
A_24_P102821	PTAFR	94.39587	74.40947	31.852428
A_23_P204304	PTPRO	38.3027	28.45494	6.8271947
A_23_P254165	RAI2	199.90884	174.7175	79.39588
A_32_P223140	RASGEF1A	465.00275	400.2592	201.76689
A_23_P124642	RASGRP1	44.7593	41.4334	16.083992
A_33_P3316953	RBFOX3	22.020144	21.96845	5.968943
A_24_P794648	RBMY2EP	12.515379	12.21913	4.9999995
A_23_P46045	RGS5	100.568275	96.74564	33.1568
A_23_P103424	RGS8	42.45246	32.37952	10.76039
A_33_P3390107	RN18S1	1485.6785	1269.542	429.34302
A_23_P83347	RNF183	44.266487	37.19701	12.887339
A_33_P3326105	RNF216	17.438139	14.18067	4.9999995
A_33_P3278877	ROBO2	41.81095	45.14235	11.778869
A_32_P4018	ROR1	136.6245	119.3928	36.93271
A_33_P3299719	RP1L1	49.8689	38.76705	14.10717
A_23_P5370	RPRM	17.73967	14.50441	4.9999995
A_33_P3251552	RSPO4	10.11803	21.94482	4.9999995
A_23_P58266	S100P	3155.3093	1361.389	245.14636
A_33_P3832341	SCARNA22	1281.5205	1080.846	521.1968
A_24_P270999	SCN4A	119.318115	58.68027	23.412725
A_23_P21063	SCN5A	19.29973	14.56349	4.9999995
A_33_P3417487	SCUBE1	1255.9583	908.1393	293.94333
A_24_P82135	SECISBP2L	542.9002	274.5824	125.4158
A_23_P21057	SEPT1	12.95718	16.06274	4.9999995

A_24_P295010	SERPINB9	1236.4465	1222.712	555.5581
A_23_P361448	SESN3	12.935589	16.15054	4.9999995
A_32_P32413	SETBP1	24.915428	16.64698	4.9999995
A_24_P940921	SETX	1395.1782	1179.328	611.643
A_33_P3702906	SF3B2	19.65971	13.12193	4.9999995
A_33_P3335177	SFRP4	16.24293	13.3001	4.9999995
A_32_P213091	SHISA9	32.53588	22.78024	4.9999995
A_23_P22761	SHOX	199.76776	95.43436	34.331226
A_23_P24616	SIAE	630.116	838.5981	149.89049
A_33_P3272260	SIRPB2	16.69374	12.11104	4.9999995
A_23_P42189	SLC17A1	11.678899	10.94915	4.9999995
A_23_P255695	SLC17A3	134.5504	116.4848	52.71851
A_23_P42004	SLC22A16	15.60968	12.0074	4.9999995
A_33_P3235841	SLC22A20	818.207	698.2045	347.166
A_33_P3281537	SLC27A3	187.64285	151.6206	41.079285
A_33_P3237210	SLC35A4	110.234314	75.58564	23.952467
A_33_P3241388	SLC4A5	25.846882	30.51217	7.3654385
A_23_P76386	SLC6A12	13.18218	15.87297	4.9999995
A_33_P3242623	SLC7A11	5558.31	6136.111	2295.233
A_23_P150343	SLN	54.35466	19.61431	5.711442
A_24_P190472	SLPI	264.157	160.9896	69.81725
A_33_P3238074	SMCR7L	23474.68	18333.35	7200.793
A_23_P382811	SNX31	16.502169	21.25393	4.9999995
A_23_P254741	SOD3	516.92773	422.5099	219.41463
A_33_P3340782	SPINK5	39.83067	34.56748	11.32581
A_23_P252817	SST	20.75686	16.16131	5.280222
A_23_P33847	SYN1	587.8486	453.3568	212.83302
A_33_P3210278	SYNE2	672.84607	489.2181	200.22624
A_24_P96897	SYT6	93.10165	107.9431	34.369316
A_23_P211345	TBX1	569.6781	582.7272	249.6256
A_24_P365365	TCF3	5252.3716	4831.618	2274.6309
A_33_P3232965	TDRD6	53.285233	40.50256	12.725171
A_23_P97826	TECTB	10.885241	11.05257	4.9999995
A_33_P3331335	TET3	18.531422	13.15533	4.9999995
A_23_P41009	TEX264	1898.9603	1442.048	745.77313
A_23_P363954	THRSP	15.81555	13.04146	4.9999995
A_33_P3336103	TIGD5	41128.297	33480.28	18448.086
A_33_P3235213	TIGIT	147.91269	125.8997	54.92036
A_23_P427645	TLL1	14.269569	12.17917	4.9999995
A_23_P322735	TMC1	36.867935	29.25614	9.470412
A_24_P787889	TMEM191B	712.9284	583.7192	300.79034
A_33_P3390723	TMEM2	294.19934	197.6524	83.35206
A_33_P3216605	TMEM63A	11.176061	11.70902	4.9999995
A_33_P3494748	TMEM65	1122.9824	321.2014	104.5423

A_24_P121956	TMEM86B	3292.0916	3920.256	1152.6221
A_33_P3296846	TMPRSS4	81.754555	54.19719	20.06273
A_33_P3216610	TMPRSS4	1239.0607	629.3239	321.17212
A_23_P211493	TMPRSS6	386.3803	394.7298	168.46912
A_23_P390518	TNFRSF11A	124.97862	103.4727	41.88632
A_33_P3354429	TRIM71	125.21012	56.64276	14.8984785
A_24_P62659	TSPAN2	10.98763	19.72767	4.9999995
A_33_P3322259	TSPY2	10.478792	19.30376	4.9999995
A_23_P422240	UROC1	33.954834	30.26026	9.92318
A_33_P3240482	VENTXP7	38.149986	22.63723	6.083512
A_32_P106376	VGLL2	44.50973	17.09081	4.9999995
A_23_P132763	VGLL3	1276.9119	1205.515	435.2563
A_23_P56578	VIT	10.248149	12.72112	4.9999995
A_33_P3277898	VSTM2A	27.428986	23.2904	6.8880258
A_23_P96331	WAS	777.02185	489.5202	227.61977
A_23_P319549	WDR65	20.509829	14.35485	4.9999995
A_33_P3252804	WEE2	36.199512	21.86339	7.081534
A_24_P237583	WFDC8	84.3789	32.58867	10.35077
A_33_P3222354	XAGE-4	17.797394	13.57633	4.9999995
A_23_P168909	ZFPM2	1528.977	1264.908	681.0875
A_24_P942743	ZFY	27.732662	19.56058	4.9999995
A_33_P3326927	ZNF19	363.49356	253.6122	122.0662
A_23_P85969	ZNF326	2410.212	1707.684	901.0171
A_33_P3242508	ZNF365	180.82373	138.0556	19.342228
A_32_P79591	ZNF665	81.090164	58.42321	23.10911
A_33_P3385775	ZNF703	1895.7935	1395.877	735.5571
A_23_P168726	ZPBP	43.477135	31.45924	11.896152

Supplementary File 2

Materials and Methods

This study was carried out in strict accordance with the recommendations in the Guide for the Care and Use of Laboratory Animals of the National Institutes of Health. The protocol was approved by the Committee on the Ethics of Animal Experiments and Human Subject Research of School of Pharmaceutical Science, Jilin University. All operations were performed under sodium pentobarbital anesthesia, and all efforts were made to minimize suffering. The participants have provided the written informed consent to participate in this study.

Isolation and characterization of human UC-MSCs

Human umbilical cords were obtained after caesarean section of normal pregnancies at First Hospital of Jilin University. The UC-MSCs were extracted as the reference [1]. In brief, the collected umbilical cords were washed in phosphate-buffered saline for 3 times, and further minced and incubated with collagenase IV (1 mg/ml, Gibco) for 4 hours, then DNase I (1 mg/ml, Gibco) for 15 min, and 0.25% trypsin (Gibco) for 15 min at 37℃. After filtration through a 40-μm cell strainer, the cells were then collected by centrifugation, and cultured in DMEM/F12 (Hyclone), containing 10% fetal bovine serum (FBS; Gibco), penicillin/streptomycin (100 U/mL to 0.1 mg/mL; Hyclone), and 2 mM L-glutamine (Gibco), in a humidified incubator with 5% CO2 at 37℃. The media were changed every other day, and the isolated UC-MSCs were passaged by a dilution of 1:3 once every 4 days or 5 days until passage 4 or 5, when a

portion of the resulting UC-MSCs was prepared for phenotypic analysis by flow cytometry. The cells were then screened for CD14, CD29, CD34, CD44, CD45, CD73, CD90, CD105, and CD166, and further differentiated into adipocytes and osteocytes as previously described [2-4].

Establishment of IGF-1 overexpressing cell lines

To generate IGF-1 overexpressing vectors, the IGF-1-coding sequences were obtained by reverse transcription PCR and further cloned into pMXs-based retroviral plasmid (Addgene). At early passage, 40,000 human UC-MSCs were transduced in 6-well culture dishes using pMX-based retroviruses as described [1, 5], to establish IGF-1 overexpressing UC-MSCs (UC-MSCs-IGF-1), and UC-MSCs infected with retrovirus containing blank pMXs vector (UC-MSCs-vector) were used as the control group in the following detections.

To assess IGF-1 expression in UC-MSCs-IGF-1, qPCR and FACS analysis were performed in our study. Besides, the effects of IGF-1 overexpression on proliferation and secretion of UC-MSCs were further evaluated in our study. UC-MSCs, UC-MSCs-vector, and UC-MSCs-IGF-1 were planted in 96-well plates at 1×10^3, respectively. Cell proliferation was detected at day 0, day 1, day 2, and day 3. The DMEM/F12 medium containing 10% FBS was used as the blank control group, and the proliferation index of each group was determined using the CCK-8 method (Dojindo, Japan) according to the manufacturer's instructions, which allowed sensitive colorimetric assays for the determination of the number of viable cells in cell

proliferation. In brief, 10 μL of CCK-8 solution was added into each well (containing 100 μL of medium), and cultured for 1 hour to 2 hours at 37 ℃. The absorbance of each group at 450 nm was detected (n=4) and it was directly proportional to the number of living cells. The proliferation index = the absorbance of experimental group − the absorbance of blank group, was used to measure cell proliferation in our study.

After 2 days of culture, the medium of each group was collected for secretory cell function analysis. Vascular endothelial growth factor (VEGF), insulin-like growth factor-1 (IGF-1), bone morphogenetic protein-7 (BMP-7), and interleukin-10 (IL-10), which were secreted by UC-MSCs and relieved kidney injury, were measured using an ELISA Kit (Abcam, United Kingdom) following the manufacturer’s instructions.

Real-time quantitative PCR

Total RNA was extracted with Trizol (Invitrogen). Then, 2 mg of RNA was reverse transcribed using RT-PCR kit (Takara) and qPCR was performed using a Thermal Cycler Dice^{TM} Real Time System and SYBR Green Premix EX Taq^{TM} (Takara). In our study, β-actin was used for Real-time quantitative PCR (qPCR) normalization and all items were measured in triplicate. All primer sequences (5’-3’) are as follows:

IGF-1 forward (F) 5’-GGTGGATGCTCTTCAGTTCGTG-3’

reverse (R) 5’-AAATGTACTTCCTTCTGGGTCTTGG-3’

Caspase 3 forward (F) 5’- GAAATTGTGGAATTGATGCGTGA -3’

reverse (R) 5’- CTACAACGATCCCCTCTGAAAAA -3’

Bax forward (F) 5’- CCCGAGAGGTCTTTTTCCGAG -3’

reverse (R) 5’- CCAGCCCATGATGGTTCTGAT -3’

Fas forward (F) 5’- TCTGGTTCTTACGTCTGTTGC -3’

reverse (R) 5’- CTGTGCAGTCCCTAGCTTTCC -3’

Bcl-2 forward (F) 5’- GGTGGGGTCATGTGTGTGG -3’

reverse (R) 5’- CGGTTCAGGTACTCAGTCATCC -3’

NCF2 forward (F) 5’- CCAGAAGCATTAACCGAGACAA -3’

reverse (R) 5’-CCTCGAAGCTGAATCAAGGC-3’

NLE2L2 forward (F) 5’- TCAGCGACGGAAAGAGTATGA-3’

reverse (R) 5’- CCACTGGTTTCTGACTGGATGT-3’

MT3 forward (F) 5’- AGACCTGCCCCTGCCCTTCT -3’

reverse (R) 5’- GAGGTGCATTTGCATCCCTC -3’

ZNF205 forward (F) 5’- CCCAGGACAAGGAGACACC-3’

reverse (R) 5’- TTAGAGCCGTGACTTAGGGGT-3’

GPX3 forward (F) 5’- AGAGCCGGGGACAAGAGAA-3’

reverse (R) 5’- ATTTGCCAGCATACTGCTTGA -3’

IER3 forward (F) 5’- CAGCCGCAGGGTTCTCTAC -3’

reverse (R) 5’- GATCTGGCAGAAGACGATGGT -3’

PPARG forward (F) 5’- GGGATCAGCTCCGTGGATCT -3’

reverse (R) 5’- TGCACTTTGGTACTCTTGAAGTT -3’

C3 forward (F) 5’- GGGGAGTCCCATGTACTCTATC-3’

reverse (R) 5’- GGAAGTCGTGGACAGTAACAG-3’

FABP4 forward (F) 5’- ACTGGGCCAGGAATTTGACG-3’

reverse (R) 5’- CTCGTGGAAGTGACGCCTT-3’

ITGA2 forward (F) 5’- CCTACAATGTTGGTCTCCCAGA-3’

reverse (R) 5’- AGTAACCAGTTGCCTTTTGGATT-3’

TGF-β1 forward (F) 5’- CTAATGGTGGAAACCCACAACG-3’

reverse (R) 5’- TATCGCCAGGAATTGTTGCTG-3’

CCR7 forward (F) 5’- TGAGGTCACGGACGATTACAT-3’

reverse (R) 5’- GTAGGCCCACGAAACAAATGAT-3’

FCER1G forward (F) 5’- AGCAGTGGTCTTGCTCTTACT -3’

reverse (R) 5’- TGCCTTTCGCACTTGGATCTT-3’

ITGB2 forward (F) 5’- TGCGTCCTCTCTCAGGAGTG-3’

reverse (R) 5’- GGTCCATGATGTCGTCAGCC-3’

C3AR1 forward (F) 5’- GTTCGTGTACCGGGAAATCTT-3’

reverse (R) 5’- CAGGCGGCTGAACAATGTTT-3’

DDR1 forward (F) 5’- GCAGCGTGTCCTAGAGCAC-3’

reverse (R) 5’- CCGCAGGTTTTCCCTGACT-3’

LRP1 forward (F) 5’- CTATCGACGCCCCTAAGACTT -3’

reverse (R) 5’- CATCGCTGGGCCTTACTCT-3’

PDGFB forward (F) 5’- CTCGATCCGCTCCTTTGATGA -3’

reverse (R) 5’- CGTTGGTGCGGTCTATGAG -3’

TNF forward (F) 5’- CCTCTCTCTAATCAGCCCTCTG -3’

reverse (R) 5’- GAGGACCTGGGAGTAGATGAG -3’

IL-6 forward (F) 5’- ACTCACCTCTTCAGAACGAATTG -3’

reverse (R) 5’- CCATCTTTGGAAGGTTCAGGTTG -3’

COX-2 forward (F) 5’- TTACAATGCTGACTATGGCTAC -3’

reverse (R) 5’- AACTGATGCGTGAAGTGCTG-3’

PI3K forward (F) 5’-TGGTTCTTGCGAAGTGAGATAG-3’

reverse (R) 5’-AAGTTGGAAGGACCACATGC-3’

AKT forward (F) 5’-TAGGCATCCCTTCCTTACAGC-3’

reverse (R) 5’- CACTGTCCCATCCGGCTTCA-3’

β-actin forward (F) 5’-CCCAGAGCAAGAGAGG-3’

reverse (R) 5’-GTCCAGACGCAGGATG- 3’

Flow cytometry

Human UC-MSCs were dissociated into single cells with trypsin, and further fixed and permeated with Fixation Buffer (BD) and Perm/Wash Buffer (BD), respectively. Finally, the cells were prepared at a concentration of 1.0×10^5 cells in 100 μL of PBS. The antibodies, including CD14 conjugated to FITC (BD), CD29 conjugated to FITC (BD), CD34 conjugated to PE (BD), CD44 conjugated to FITC (BD), CD45 conjugated to PE (BD), CD73 conjugated to APC (BD), CD90 conjugated to FITC (BD), CD105 conjugated to APC (BD), CD166 conjugated to PE (BD), and IGF-1 conjugated to APC (LSBio), were added and incubated for 30 min at 4℃. After two washes in PBS, the cells were acquired and analyzed with FACScalibur (BD Bioscience).

Preparation and treatment of AKI model

Male node rats, weighing 250 g to 300 g, were purchased from Vital River Laboratories (Beijing, China). All rats were housed in the room with constant temperature and a 12 hours dark-12 hours light cycle. The node rats were fed a standard diet. The method used to prepare or treat the AKI model was similar to our previous research [3, 4]. In brief, animals were divided into six groups (six rats per group), namely, normal group, AKI model group, positive drug group, UC-MSCs group, UC-MSCs-vector group, and UC-MSCs-IGF-1 group. To generate a rat model of AKI for testing the UC-MSCs-IGF-1 in vivo, the model was induced by gentamicin (140 mg/kg/day for 7 days, i.p.) in rats. At day 8, the stem cells were administered by intravenous injection of 3.3×10^6 cells/kg, whereas dexamethasone (0.08 mg/kg) was used as a positive control drug during the 7-day therapy. The normal group and AKI model group did not receive any treatment.

Evaluation of kidney function

In our study, all animals were sacrificed on day 15. To determine the biochemical variables (NAG, LZM, creatine and urea nitrogen) using a Biochemistry Autoanalyzer, blood and urine were collected on days 8 (before treatment) and 15, respectively. For histological analysis, kidney tissue samples of each group were fixed with formalin, embedded in paraffin, sectioned to 5 μm-thick, and processed by hematoxylin and eosin (H&E) staining. Then the slides were reviewed blindly and scored with a

semiquantitative scale evaluating changes found in AKI as the reference [6]. For each kidney, 100 cortical tubules from at least 10 different areas were scored, and avoid repeated scoring of different convolutions of the same tubule. The average histological score was used to valuate kidney tissue morphology. Higher scores represented more severe damage.

Analysis of cell apoptosis

Apoptosis in the kidney tissue was evaluated by enzymatic labeling of DNA strand breaks using terminal deoxynucleotidyl transferase-mediated deoxyuridine triphosphate nick end-labeling (TUNEL) assay kit (KeyGEN) according to the manufacturer's instructions. The expression levels of apoptotic genes (Caspase 3, Bax, and Fas) and anti-apoptotic gene (Bcl-2) in the kidney tissues of each group were further detected using real-time qPCR, and some apoptotic genes were also confirmed with immunohistochemistry.

Immunohistochemistry

The cells or kidney tissues were fixed in 4% paraformaldehyde in PBS. The kidney tissues of each group were embedded in paraffin and sliced into 5 μm-thick sections. For immunohistochemistry, the primary antibodies used were anti-Caspase 3 (1:100, Abcam), anti-Bcl-2 (1:100, Abcam), and anti-human nucleus antigen (hNA, 1:500, Millipore) polyclonal antibodies. After 12 hours of incubation at 4 ℃, the samples were washed three times with PBS and processed using an SABC kit and DAB

solution. Finally, the sections were observed using Axio Scope A1 (Zeiss, Germany) with AxioCAM MRc5 (Zeiss), and processed with AxioVision software (Zeiss). The color intensity was measured using Motic Image Advanced 3.2.

Microarray analysis

Total RNA was extracted from UC-MSCs, UC-MSCs-vector and UC-MSCs-IGF-1 using Trizol (Invitrogen) and the RNeasy kit (Qiagen). Samples were amplified and labeled using a Aglient Quick Amp Labing Kit. Array hybridization was analyzed with the Aglient Hybridization System and followed by washing with the Aglient wash buffer kit. The Aglient DNA microarray scanner was used for array scanning. Genes that have values greater than or equal to lower cut-off: 50.0 in 2 out of two samples were chosen for data analysis. Differentially expressed genes were identified through Fold Change filtering. Pathway Analysis and GO analysis were applied to determine the roles of these differentially expressed genes played in these biological pathways or GO terms. Finally, Hierarchical Clustering was performed to show distinguishable gene expression profiling among samples.

Knockdown of IGF-1 in UC-MSCs-IGF-1 using siRNA

For small interfering RNA (siRNA)-mediated knockdown of IGF-1, UC-MSCs-IGF-1 were transfected with IGF-1-siRNA (UC-MSCs-siRNA) (Santa), while the cell transfected with nontargeting siRNA were used as the control group (UC-MSCs

-control). The detailed operation was performed according to the manufacturer’s instructions.

Anti-oxidation capacity assay

After the therapy, the levels of the ROS, as well as some antioxidants (SOD, eNOS and HO-1) in injured kidney tissues of each group were detected with kits purchased from Nanjing Jiancheng Bioengineering Institute. Kidney tissues (200 mg) were homogenized with PBS solution and the homogenate was centrifuged at 3000 rpm for 20 min. The supernatant was collected for further detection and the detailed operations were performed according to the manufacturer’s instructions.

To further evaluate the anti-oxidation capacity of UC-MSCs-IGF-1 in vitro, a co-culture system was established using trans-well. UC-MSCs, UC-MSCs-vector and UC-MSCs-IGF-1 were seeded on the upper side of a porous polycarbonate membrane (pore size: 8 μm; Euroclone SPA) in co-culture with renal tubular epithelial cell line，HK2 cells, respectively. The HK-2 cells (ATCC, USA) were seeded at 1×10^5 cells/well in a 24-well plate. Keep the ratio of HK2 cells and UC-MSCs at 5:1. The cells were incubated with DMEM/F12 (Hyclone) plus 10% FBS (Gibco) in the co-culture system, and exposed to 30 μM H_2O_2 for 1 day and 2 days respectively. Finally, proliferation index of HK2 cells in each group was detected with CCK-8, and the HK2 cells co-cultured with noting were used as the control group.

Anti-inflammatory potential assay

The inflammatory components in the kidney tissues of each group were detected with ELISA. Similar to anti-oxidation capacity assay, the supernatant from kidney tissue was collected for the detection. Herein, monocyte/macrophage chemotactic protein-1 (MCP-1), Macrophage inflammatory protein 2 (MIP-2), regulated upon activation normal T cell expressed and presumably secreted (RANTES), and transforming growth factor-β (TGF-β) and were examined for evaluation of inflammatory reaction.

In addition, to further detect the anti-inflammatory potential of UC-MSCs, UC-MSCs-vector and UC-MSCs-IGF-1, a co-culture system was established using trans-well. Similar to anti-oxidation capacity assay, UC-MSCs, UC-MSCs-vector and UC-MSCs-IGF-1 were seeded on the upper side of a porous polycarbonate membrane in co-culture with murine macrophage RAW264.7 cell line (ATCC, USA) respectively. The RAW264.7 cells were seeded at 1×10^5 cells/well in a 24-well plate. Keep the ratio of RAW264.7 cells and UC-MSCs at 5:1. The cells were incubated with DMEM/F12 (Hyclone) plus 10% FBS (Gibco) in the co-culture system, and exposed to lipopolysaccharide (100 ng/ml, Sigma) for 24 hours. Finally, the expression of genes associated with inflammatory response (TNF, IL-6 and COX-2) in RAW264.7 cells of each co-culture group was detected with qPCR, and the RAW264.7 cells co-cultured with noting were used as the control group.

Cell migration assay

To evaluate the effect of IGF-1 overexpression on migratory ability of UC-MSCs in vivo, anti-human nucleus antigen (hNA, Millipore) was used to detect the location of

human mesenchymal stem cells in kidney tissue of each group with immunohistochemistry. The number of hNA-positive cells was further counted in ten fields for each group, and the average value was used to evaluate the migratory ability of UC-MSCs, UC-MSCs-vector and UC-MSCs-IGF-1.

To investigate the influence of cell proliferation on the migration, mitomycin C was used to block proliferation as the reference [3]. The migration of UC-MSCs was still measured using Tran-swell plates as the previous study [3]. In brief, UC-MSCs were seeded on the upper side of a porous polycarbonate membrane in co-culture with HK2 cells. HK2 cells were seeded at 1×10^5 cells/well in a 24-well plate. After 24 hours, the cells were incubated with DMEM/F12 plus 2% FBS alone or in the presence of 5 μM cisplatin for 6 hours. After cisplatin removal, the HK2 cells were washed and co-cultured with UC-MSCs. After 36 h, the cells at the upper side of the filter (unmigrated cells) were mechanically removed. Cells that had migrated to the lower side of the filter were fixed for 30 min in 4% paraformaldehyde and further stained with crystal violet. The number of cells in ten random fields was counted for each filter.

Statistical analysis

The results were expressed as means ± SEM, and statistical analysis was performed using SPSS 17.0. The differences among groups were analyzed by one-way ANOVA followed by t-test. $P < 0.05$ was considered statistically significant.

References

1. Cai J, Li W, Su H, *et al.* Generation of human induced pluripotent stem cells from umbilical cord matrix and amniotic membrane mesenchymal cells. *J Biol Chem* 2010; **285:** 11227-11234.

2. Cheng K, Rai P, Plagov A, *et al.* Transplantation of bone marrow-derived MSCs improves cisplatinum-induced renal injury through paracrine mechanisms. *Exp Mol Pathol* 2013; **94:** 466-473.

3. Liu P, Feng Y, Dong C, *et al.* Administration of BMSCs with Muscone in Rats with Gentamicin-Induced AKI Improves Their Therapeutic Efficacy. *PloS one* 2014; **9:** e97123.

4. Liu P, Feng Y, Dong C, *et al.* Study on therapeutic action of bone marrow derived mesenchymal stem cell combined with vitamin E against acute kidney injury in rats. *Life Sci* 2013; **92:** 829-837.

5. Esteban MA, Wang T, Qin B, *et al.* Vitamin C enhances the generation of mouse and human induced pluripotent stem cells. *Cell Stem Cell* 2010; **6:** 71-79.

6. Paller MS, Hoidal JR, Ferris TF. Oxygen free radicals in ischemic acute renal failure in the rat. *J Clin Invest* 1984; **74:** 1156-1164.

Supplementary Figure 1

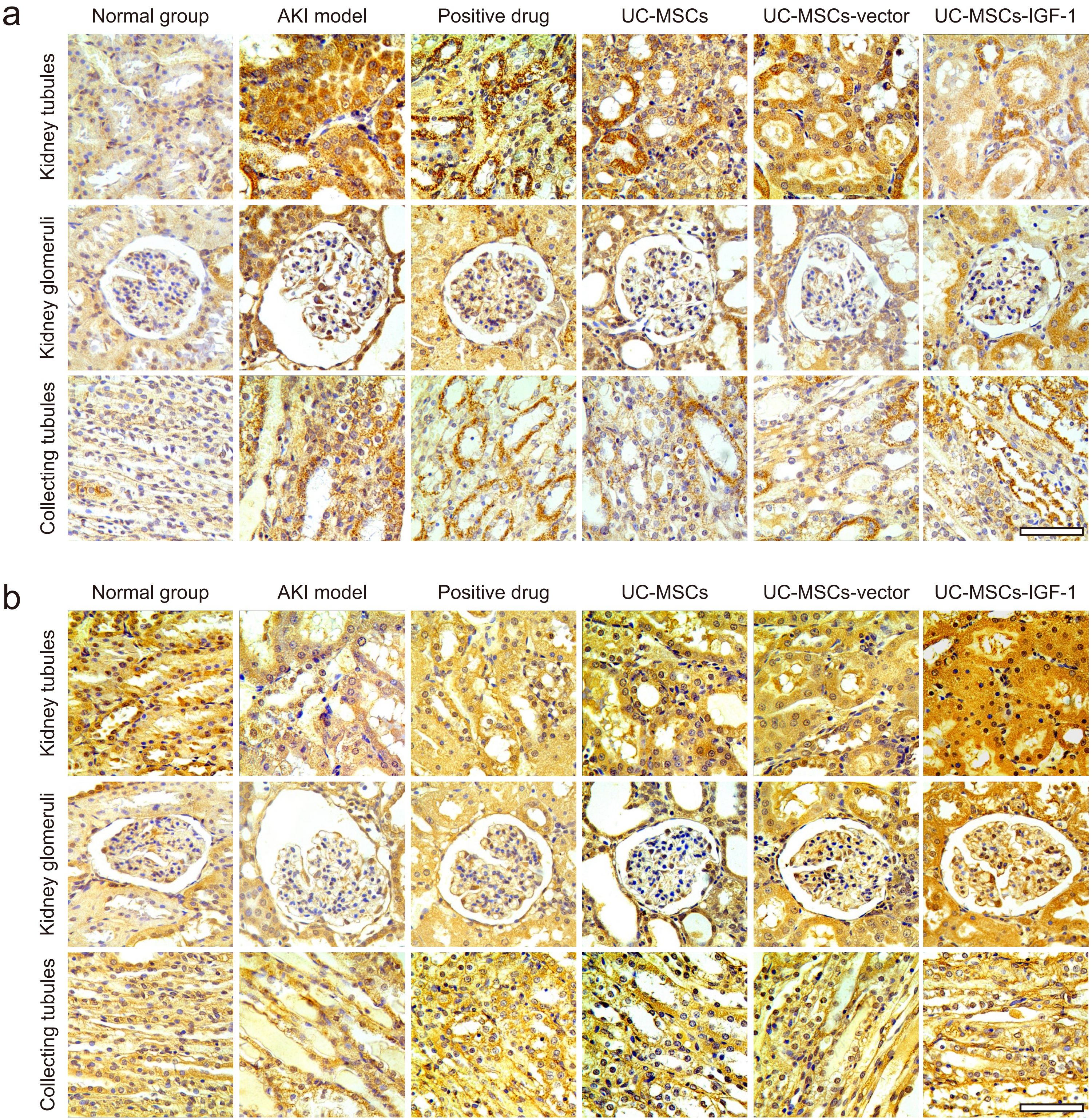

Supplementary Figure 2

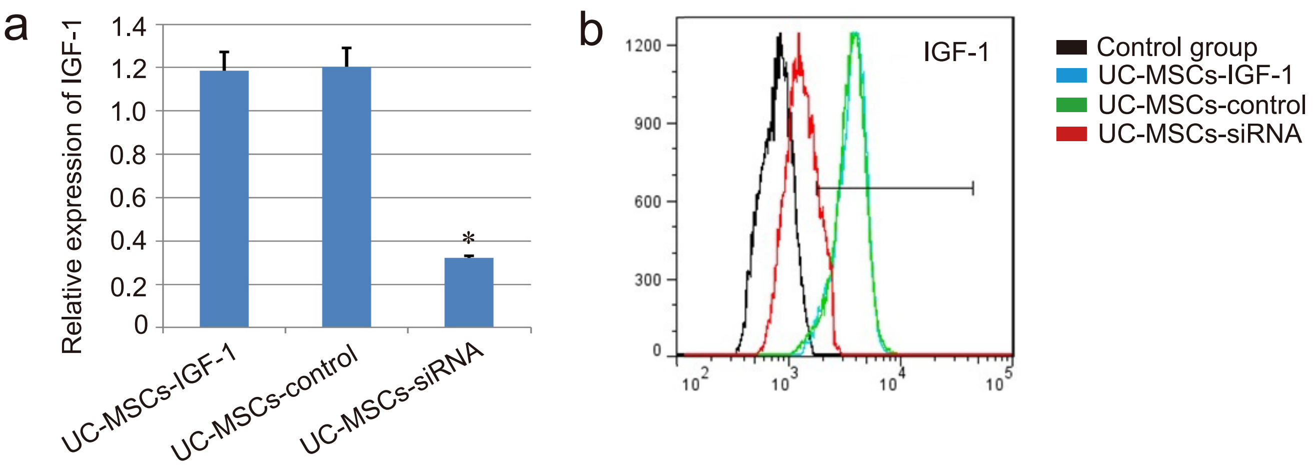

Supplementary Figure 3

a

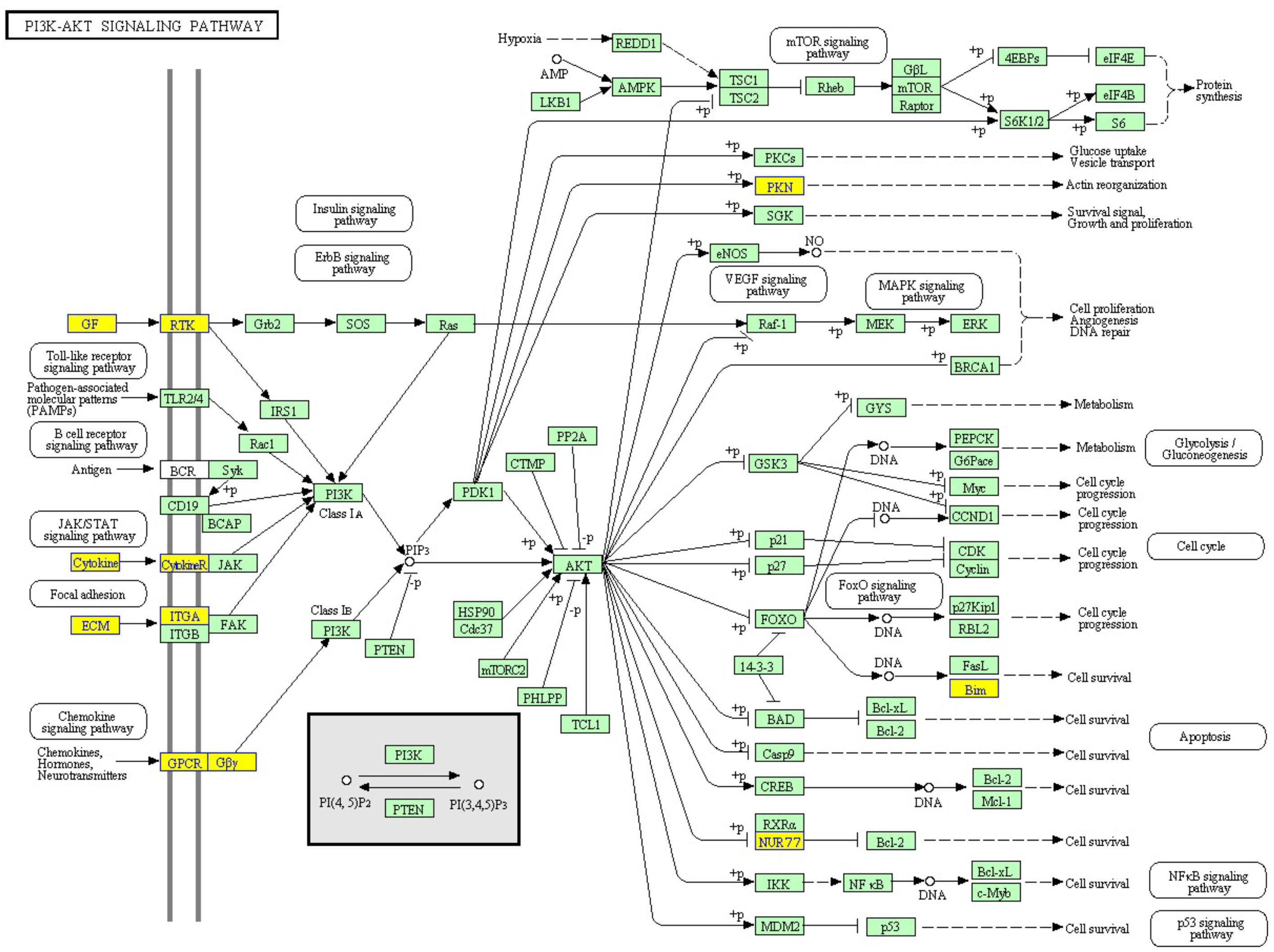

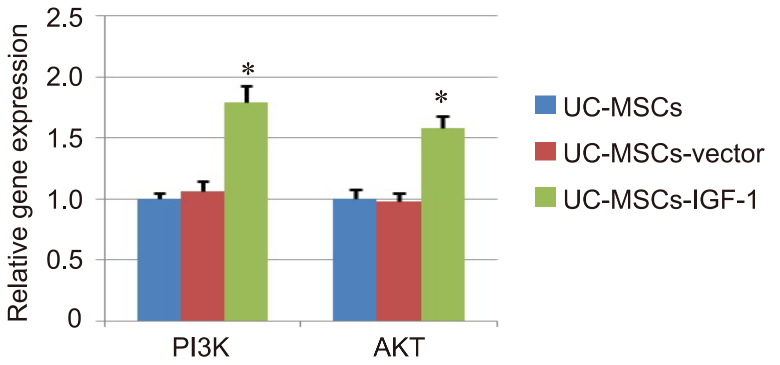

b

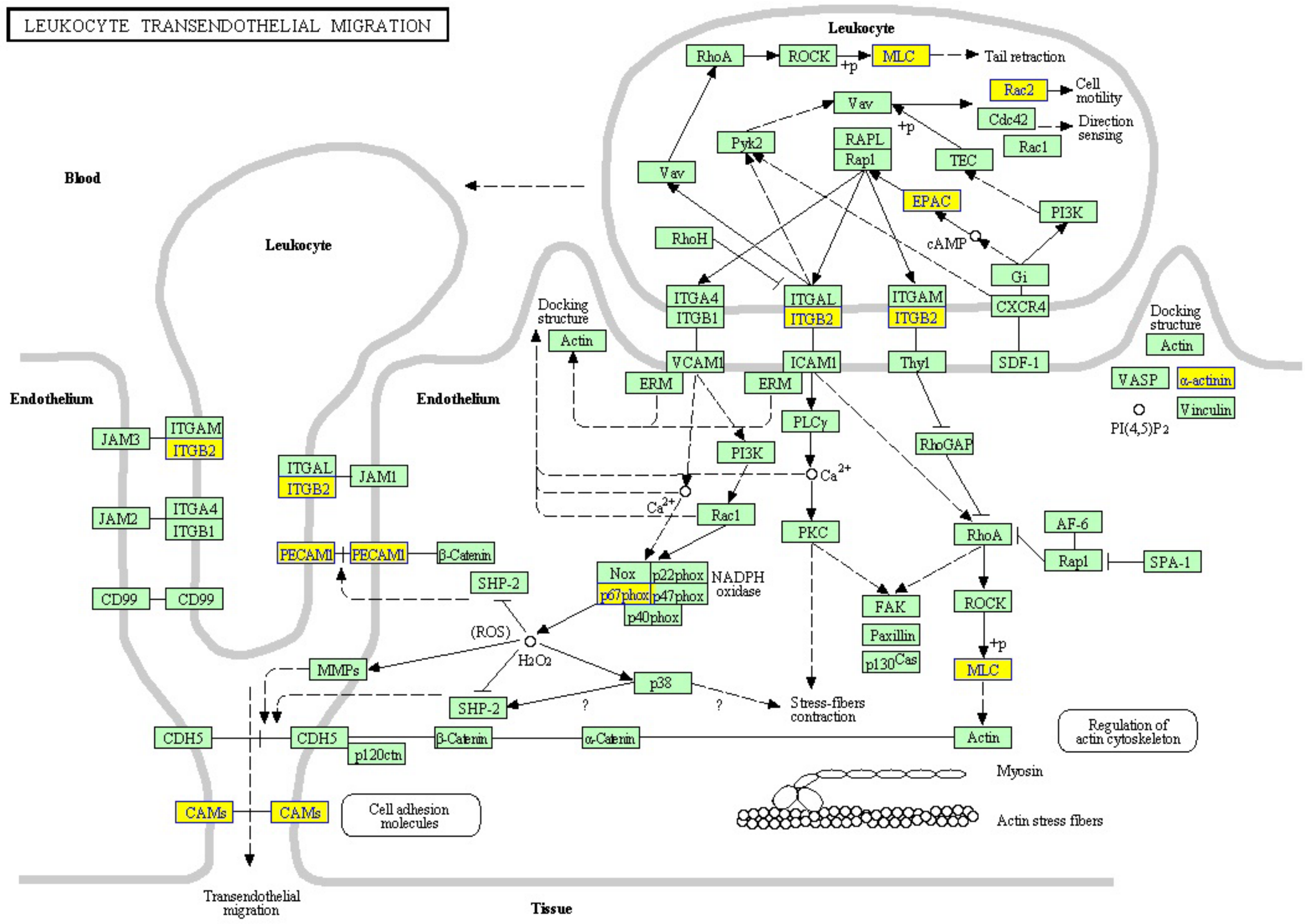

Supplementary Figure 4

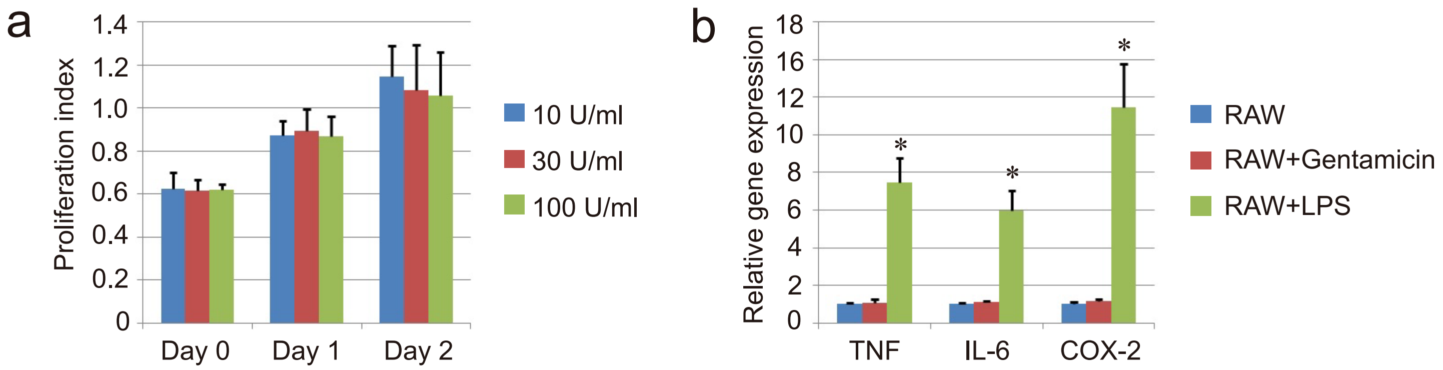

Supplementary Table 1. Change of biochemical parameters in serum or urine during the therapy process

	Characteristics of AKI model in urine or serum				Biochemical parameters in urine after therapy			Biochemical parameters in serum after therapy		
	NAG(U/L)	LZM(U/ml)	Creatine(μM)	UN(mM)	NAG(U/L)	LZM(U/ml)	UN(mM)	NAG(U/L)	Creatine(μM)	UN(mM)
normal group	14.5±1.4	33.5±2.7	22.7±3.4	10.5±1.3	18.2±1.5	36.0±3.7	38.9±3.8	18.1±2.0	21.0±4.0	8.3±1.6
AKI model	37.5±4.0*	177.5±13.2*	70.5±5.7*	27.2±4.0*	44.3±6.1	247.5±27.0	15.3±2.0	66.4±8.8	81.6±9.2	34.5±5.0
positive drug					29.9±4.6*	237.0±24.2	21.9±2.9*	34.9±6.8*	43.5±8.8*	31.3±4.0
UC-MSCs					27.7±2.7*	220.3±11.8	24.3±2.8*	44.5±5.7*	44.8±7.4*	31.6±5.7
UC-MSCs-vector					28.3±6.3*	224.9±23.1	26.7±2.5*	44.2±4.8*	47.9±4.6*	30.7±6.9
UC-MSCs-IGF-1					26.4±3.0*	200.5±20.7*#	34.65±4.0*#	41.2±7.8*	34.4±5.1*#	24.1±3.6 *#

Supplementary Information

Supplementary File 1. The raw data of heatmap in Figure 5a. Compared with normal UC-MSCs and UC-MSCs-vector, the up-regulated and down-regulated genes in UC-MSCs-IGF-1 were shown herein respectively.

Supplementary File 2. The detailed information about the materials and methods.

Supplementary Figure 1. Expression of Caspase 3 and Bcl-2 in the kidney tissues of each group. The expression levels of apoptotic gene (Caspase 3, Supplementary Figure 1a) and anti-apoptotic gene (Bcl-2, Supplementary Figure 1b) in the kidney tissue were detected using immunohistochemistry. A representative in the kidney tubules, kidney glomeruli, and collecting tubules close to the average level of each group is shown for each group. Scale bar corresponds to 50 μm.

Supplementary Figure 2. Analysis of effect of IGF-1-siRNA on IGF-1 expression in UC-MSCs-IGF-1 by qPCR (Supplementary Figure 2a) and FACS (Supplementary Figure 2b). * $P<0.05$ compared with the UC-MSCs-IGF-1 group and UC-MSCs-control group respectively.

Supplementary Figure 3. Signal pathway analysis of the effects of IGF-1 overexpression in UC-MSCs through RNA microarray. a. PI3K-AKT signaling pathway. The expression of PI3K and AKT was further detected with qPCR. The level

of gene expression in UC-MSCs was regarded as 1.0. * P<0.05 compared with the normal UC-MSCs group and UC-MSCs-vector group respectively. b. Leukocyte transendothelial migration pathway. “Yellow” indicated the upregulated genes, and “cyan” indicated the genes without significant changing.

Supplementary Figure 4. Biological effects of gentamicin on HK-2 cells and RAW264.7 cells. a. Effect of gentamicin on HK-2 cells proliferation. The proliferation index was evaluated with CCK-8 method, and results are expressed as mean ± SEM. b. Function of gentamicin (100U/ml) to induce inflammation in RAW264.7 cells. The expression of TNF, IL-6 and COX-2 was detected using qPCR. The level of gene expression in RAW264.7 cells without any treatment (RAW) was regarded as 1.0, then gene expression in RAW264.7 treated with gentamicin (RAW+gentamicin) and LPS (RAW+LPS) was further performed. * P<0.05 compared with the normal RAW group and RAW+gentamicin group respectively.

Supplementary Table 1. Change in biochemical variables in serum or urine during the therapy process. To assay the characteristics of rat AKI model, NAZ and LAM in urine, as well as creatinine and urea nitrogen (UN) in serum, were detected on day 7. *P <0.05 compared with the normal group. To analyze the therapeutic effect, urine and serum samples of each group were collected from each group on day 15 after animals were sacrificed. The levels of UN, NAZ, and LAM in urine, as well as creatinine, UN, and NAG in serum, were measured. *P < 0.05 compared with the

model group; #$P < 0.05$ compared with the normal UC-MSCs group and UC-MSCs-vector group respectively.
